# Bamdam: a post-mapping authentication toolkit for ancient metagenomics

**DOI:** 10.1186/s13059-025-03879-x

**Published:** 2025-12-05

**Authors:** Bianca De Sanctis, Cade Mirchandani, Haoran Dong, Ruairidh Macleod, Russell Corbett-Detig, Yucheng Wang

**Affiliations:** 1 Genomics Institute, University of California Santa Cruz, Santa Cruz, USA; 2 Department of Ecology and Evolutionary Biology, University of California Santa Cruz, Santa Cruz, USA; 3 Centre for Ancient Environmental Genomics, Globe Institute, University of Copenhagen, 1350 Copenhagen, Denmark; 4 Department of Biomedical Engineering, University of California Santa Cruz, Santa Cruz, USA; 5 Key Laboratory of Western China’s Environmental Science (Ministry of Education), College of Earth and Environmental Sciences, Lanzhou University, Lanzhou, China; 6 Group of Alpine Paleoecology and Human Adaptation (ALPHA), State Key Laboratory of Tibetan Plateau Earth System, Resources and Environment (TPESRE), Institute of Tibetan Plateau Research, Chinese Academy of Sciences, 100101 Beijing, China; 7 Department of Genetics, University of Cambridge, Cambridge, CB2 3EH UK

**Keywords:** Metagenomics, Environmental DNA, Ancient DNA, Read complexity, Authentication

## Abstract

Ancient metagenomic studies using capture or shotgun sequencing often perform pairwise alignment of individual reads against large reference databases followed by lowest common ancestor assignment for taxonomic identification. Here, we present bamdam, a lightweight post-mapping, post lowest common ancestor toolkit for eukaryotic or microbial metagenomics. Bamdam can shrink large metagenomics bam files, often by a factor of 10x or more, while retaining all informative reads and alignments, compute a suite of authentication metrics for each taxonomic node including *k*-mer duplicity, postmortem damage, and mean read complexity, and generate various visualizations including multi-sample deamination plots and damage-colored interactive Krona plots.

**Supplementary Information:**

The online version contains supplementary material available at 10.1186/s13059-025-03879-x.

## Background

Metagenomic analysis of environmental DNA (eDNA) is a powerful tool that has been increasingly used to study the genetic makeup of an entire ecosystem. Environmental DNA can be isolated and analyzed from a wide variety of sources, including soil, water, ice, permafrost, and even air, and the ability to sequence ancient eDNA is extending ever further into the past [1–6]. Metagenomic studies make use of several different sequencing approaches, including shotgun sequencing, hybridization capture, or metabarcoding. While metabarcoding targets and amplifies sequences from a small genomic region—allowing for high sensitivity in taxa identification—it prevents the use of metrics such as postmortem damage analysis, which is critical for authentication if the DNA is suspected to be ancient. In contrast, capture and shotgun sequencing recover reads from larger genomic regions, such as entire mitochondrial genomes or indiscriminately from the whole genome, and thus allow for the analysis of postmortem damage signals [7]. As sequencing costs continue to decline, shotgun sequencing and capture methods are increasingly favored over metabarcoding in ancient eDNA studies [6–8].

This shift has been accompanied by the development of dedicated modern or ancient eDNA shotgun sequencing taxonomic assignment methods [8–11]. To identify taxa represented in metagenomic samples, one can use *k*-mer based methods such as KrakenUniq [12], Kaiju [13] or Kraken2 [14], heuristic methods such as MEGAN, Centrifuge or mmseqs2 [15–17], or pairwise alignment methods such as bowtie2 or bwa combined with software to assign reads to their lowest common ancestor (LCA) in a reference taxonomy, such as ngsLCA or sam2lca [18–21]. At the same time, there is a growing consensus in the community that rigorous authentication metrics should be employed to validate that the taxa identified are not lab, database, or in the case of ancient DNA, modern environmental contaminants [22–29]. Authentication might include postmortem damage, number of unique *k*-mers, read lengths, edit distances, and even phylogenetic placement to confirm the presence of expected haplogroups for taxa with sufficient reads. Since postmortem damage is assessed by counting C-to-T and G-to-A transitions from the reference, its calculation requires that reads have been aligned to references, which is not typically the case with any of the aforementioned *k*-mer based and heuristic methods. On the other hand, pairwise alignment methods can be time, storage, and RAM-intensive when mapping against large reference databases, which is normally required to avoid bias introduced by skewed representations of selected reference genomes as much as possible [12].

Some workflows mitigate these computational challenges by combining the scalability of *k*-mer-based methods with targeted full sequence alignment against high scoring nodes. For example, the aMeta workflow [27] uses KrakenUniq to first identify community composition, then uses the identified taxa to build a custom MALT reference database for mapping. This two-step process allows the early exclusion of some potential database contaminants beforehand using KrakenUniq’s unique *k*-mers. Furthermore, aMeta employs several authentication metrics after mapping such as postmortem damage and read length distributions. MALT, or Megan Alignment Tool, is a fast and commonly used metagenomic aligner with a lowest common ancestor step. It is used in aMeta and also forms the core aligner in the HOPS pipeline [30]. However, the KrakenUniq screening step in aMeta is necessary to reduce database size because MALT cannot map against massive databases. For example, MALT would require 3.5TB of RAM to map against the full NCBI nt [27]. The nf-core eager workflow [31] similarly relies on MALT and HOPS and therefore cannot easily map against a large reference database.

Conventional pairwise alignment tools such as Bowtie2 or BWA are often preferred for the mapping step, at least until more specialized metagenomic solutions become available [18, 19]. Compared to MALT, Bowtie2 is faster, more memory-efficient, and outputs alignments in the standard bam format, which integrates seamlessly with widely used tools like samtools [27, 32]. MALT, in contrast, produces alignments in the special RMA6 format. However, because general-purpose aligners like Bowtie2 and BWA are not designed for metagenomics, they require a separate lowest common ancestor (LCA) assignment and authentication step. To this end, two similar LCA methods for the bam format have been developed, sam2lca and ngsLCA [20, 21]. Both rely on the same style of taxonomy files. According to the aMeta paper, “Bowtie2 + sam2lca should entirely replace MALT while delivering alignments in bioinformatics-friendly SAM-format” [27]. The combination of pairwise alignment with bowtie2 and ngsLCA, also often paired with postmortem damage estimation using metaDMG [33], is implemented in the Holi pipeline [6–8, 34].

However, this all-vs-all mapping strategy commonly yields false positive taxonomic assignments. Filtering for damage alone is insufficient; for instance, Oskolkov et al. [29] recently showed extensive microbial contamination in widely used reference genomes for ancient metagenomics, leading to misidentification of ancient taxa, even after filtering for postmortem damage, when authentic ancient microbial reads map to contaminated eukaryotic references. A need for more stringent authentication after the LCA step remains. Here we present a post-mapping, post lowest common ancestor authentication toolkit, which we call bamdam, designed for the final stage of the Holi pipeline (e.g. after ngsLCA or metaDMG) [34], or for after the bowtie2 step in aMeta in combination with ngsLCA, or indeed any eukaryotic or microbial metagenomics workflow which creates a bam file and an LCA assignment file [20].

## Results

The bamdam toolkit (a) provides customizable filtering for bam files using LCA information, shrinking bam file size by 10x or more while retaining all phylogenetically informative alignments, (b) computes a suite of authentication metrics for each taxonomic node such as the number of unique *k*-mers, mean DUST score (mean read complexity), postmortem damage and more, and (c) allows extraction of taxa-specific reads for downstream analyses, and (d) offers visualization of authentication metrics such as read length distributions, multi-sample deamination plots, and damage-colored interactive Krona plots [35].

Bamdam can be run on any read-sorted bam with an associated lca text file containing read names and taxonomic paths in the same order as the bam, allowing users the flexibility to filter as they wish. The lca file format is direct output from ngsLCA [20] or, because it is a text file with a standard format, could also be derived from sam2lca output [21] or other methods. Bamdam is also compatible with metaDMG [33], related software which uses a bam file and reference taxonomy-associated files to model postmortem damage per taxonomic node. Bamdam processes bam and lca files line by line, skipping between them without loading the entire file into memory. Regardless of input file size, this lightweight implementation uses minimal RAM and can usually run on a laptop in minutes to hours. Bamdam is open access and available at https://github.com/bdesanctis/bamdam/.

### Software description

**Fig. 1:**
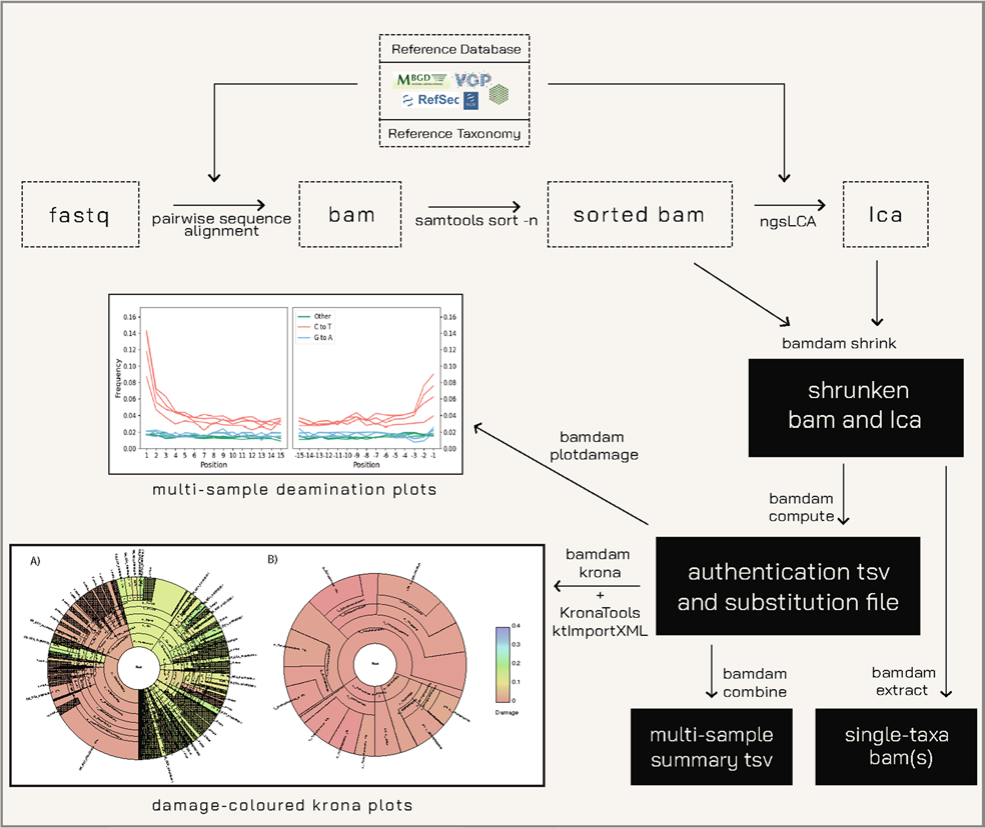
Bamdam workflow for a metagenomics experiment. After using ngsLCA for LCA assignment for individual reads, bamdam shrink is used to reduce bam file sizes up to several orders of magnitude. Next, bamdam shrink and compute are used to calculate authentication statistics such as *k*-mer duplicity, read complexity, and postmortem damage. Multi-sample, damage-coloured krona plots made with bamdam krona and ktImportXML from KronaTools [35] can be useful visualizations, especially for large metagenomics experiments. Example bamdam krona output shown is (**A**) a sample containing authentic ancient DNA and (**B**) a negative control. Bamdam extract is used to obtain reads assigned to a single taxonomic node. Bamdam plotdamage is for plotting postmortem damage frequency for a specific taxa using the nucleotide substitution frequency file, potentially from multiple samples at once (the example in this figure is for microbial samples prepared with single-stranded library prep, and which have a high baseline divergence from their closest reference). Bamdam combine can combine multiple bamdam tsv files into one multi-sample summary file. A further accessory function to plot read length distributions and mismatch plots is detailed in the manuscript and on the github

A typical workflow using bamdam is organized as in Fig. 1. One must first run the shrink function on every sample separately, and then the compute function on every sample separately. This will produce per sample a smaller lca file, a smaller bam file, a tsv file containing authentication metrics per taxonomic node, and a text file containing substitution frequencies per taxonomic node. The output of these functions can then be fed into the combine, plotdamage, extract, plotbaminfo and krona functions, most of which can optionally take as input multiple samples at once.

*Bamdam shrink*. Working with bam files mapped to large reference databases can be difficult because they are the bam files themselves are often massive in size, reaching hundreds of gigabytes each and creating storage and archiving issues in projects with many samples. In order to obtain an accurate LCA assignment, reads are typically allowed to align to at least thousands of reference genomes in a process often called competitive mapping (eg [6]). One consequence of this is that ancient metagenomic alignment files are not often deposited into public databases, despite the fact that reproducing them is computationally expensive and bam files are the main resources of data archiving for other types of genomic studies. Because studies often consider and report taxa only up to the genus or family level [6–8, 36], a large proportion of the alignments in each bam file are irrelevant to the study results. In particular, the majority of the alignments belong to reads which map to such diverse references that their LCA assignment is phylogenetically uninformative (e.g. reads assigned to “Metazoa”). Bamdam shrink takes as input a bam file and an lca file, and outputs a much smaller bam file and lca file. This lossy compression strategy greatly reduces storage needs, allows the user to interact with much smaller files for downstream tasks, eases the public sharing of data, and makes the authentication step of bamdam much faster.

To perform the shrink function, bamdam runs through the lca file twice: once to determine which taxonomic nodes pass filtering criteria, and again to extract reads assigned to those nodes. This initial filtering removes reads in the lca file assigned to taxonomic nodes which are above the taxonomic rank threshold (“family” by default, but configurable) or which have too few reads assigned to them (5 reads by default). In addition to these flags, the shrink step is configurable in several ways. First, the user may manually pre-filter their lca file beforehand, as long as reads are kept in the same order – for example, one might wish to remove all bacterial reads before running bamdam. Second, the shrink step includes an option to exclude a custom, user-defined list of taxonomic nodes from the output shrunken bam and lca files, in which case the reads are still aggregated up the taxonomy after these reads are excluded. For example, it might sometimes be desirable to remove all taxonomic nodes from the samples which were found in the negative controls as a stringent decontamination step. In this case the user would run bamdam shrink first on the controls, extract the control taxonomic node IDs, and pass these into bamdam shrink as tax IDs to exclude for the samples. Regardless of whether the user excludes nodes or not, once the filtered lca file has been written, the bam will then be filtered to only retain alignments associated to reads in the filtered lca file, and with more than a user-defined similarity (the number of matches to the reference divided by the read length; 0.9 by default). This yields a shrunken output bam file. The output bam can also optionally be annotated with PMD (postmortem-damage) scores as from PMDTools (off by default), which measure the probability of each individual alignment being damaged, as defined by the model in the original paper [37]. Running shrink is required before running compute.

*Bamdam compute* processes the bam and lca files iteratively to output two files: a tsv containing authentication metrics and a nucleotide substitution frequency text file. Both output files have one row per taxonomic node. The tsv file contains all of the authentication metrics and orders the nodes by total assigned reads, and the substitution text file stores a list of nucleotide substitution frequencies for each taxonomic node for damage plotting. Within bamdam compute, information is stored for each taxonomic node, and each new read will update the information for its assigned taxonomic node, as well as aggregate to those nodes above it. Output per taxonomic node in the tsv file includes: the taxon name, the taxon id, total reads assigned to that node or below, *k*-mer duplicity, mean DUST score, C-to-T frequency on the 5’ end, C-to-T or G-to-A frequency on the 3’ end (depending on the “stranded” argument, i.e. whether the sequencing library was double or single stranded), the mean read length, the average nucleotide identity, the average GC content of the reads, the average GC content of the associated reference regions, a HyperLogLog estimate for the number of unique *k*-mers, the number of reads assigned to exactly that node (‘unaggregated’), and the full taxonomic path. Except for the number of unaggregated reads, all statistics for a taxonomic node are calculated in an aggregated way, across the entire set of reads assigned to that node or any of its descendants. In this way, a read assigned to a species-level node also contributes to the statistics for its genus, but a read assigned to a genus-level node will not contribute to any statistics for any of its descendant species.

Many false positive taxonomic assignments involve reads which align only to one small region of a reference genome(s), rather than being approximately uniformly distributed (e.g. see [38]). This can occur from reference genome contamination, erroneous alignments, over-amplification, horizontal gene transfer or lab contamination. However, directly calculating breadth of coverage in this context is challenging, as reference databases can be terabyte-scale and reads can align to thousands of genomes, so calculating breadth of coverage per reference genome can be less meaningful or hard to interpret in a metagenomic context. Instead, inspired by KrakenUniq [12], bamdam calculates the number of unique and total *k*-mers per taxa. The number of unique *k*-mers has been shown to be useful as an authentication metric, as has the number of unique *k*-mers divided by the read count [27, 39, 40]. The unique *k*-mer counting is done using the fast HyperLogLog cardinality estimation algorithm to minimize RAM consumption [12, 41]. In bamdam, there is a fixed error threshold of $1\%$ for unique *k*-mer counting with HyperLogLog. We also calculate the *k*-mer duplicity, defined as the number of total *k*-mers divided by the number of unique *k*-mers among all the reads at or below a taxonomic node. In other words, duplicity is the average number of times a *k*-mer (or its reverse complement) has been seen in all reads assigned to a taxonomic node or underneath. Depending on coverage, *k*-mer duplicity can be (much) higher in capture sequencing data, or if only short fragments are available in the reference database, so that the number of reads assigned to a taxa is sufficient to cover the reference region more than once. Otherwise, a *k*-mer duplicity much higher than 1, or a relatively low number of unique *k*-mers, can signal a false positive, or at least that a closer look at the reads assigned to a taxonomic node is required. Bamdam accounts for reverse complements in all *k*-mer calculations.

Next, we introduce mean read complexity per taxonomic node as a post-mapping authentication metric. Sequences with low complexity may include those from genuine modern or ancient environmental taxa, laboratory amplicons, sequencing errors, poly-A or poly-T reads, and/or laboratory contaminants. No matter the origin, these low complexity reads are prone to map to similarly low-complexity regions in reference genomes, and may even carry damage signals if they originated from a genuine ancient source. If the true reference genome is not in the reference database, or the reference genome is present but does not contain these regions – because low-complexity regions are especially difficult to assemble [42] – these reads may map elsewhere. Paradoxically, these reads may then be more likely to spuriously map to high-quality reference genomes, as high-quality assemblies are more likely to contain repetitive and low complexity regions. Taxonomic nodes for which read sets have a low overall mean read complexity are thus more likely to be artifacts. This issue may then become further exacerbated over time as we sequence and include more high-quality reference genomes in our reference databases. We implement a complexity metric, DUST score [43], which measures the complexity of 3-mers within each read, and is scaled from 0 to 100, where higher scores indicate lower complexity (e.g., a score of 100 for TTTTTTTTTT). Our scaling matches the Prinseq DUST implementation [44], which notably differs from the sga implementation and the original DUST formulation [43, 45]. DUST scores are commonly used in pre-mapping quality control to remove low-complexity reads (e.g., [44, 45]), and some curated databases are DUST-masked, both usually at a maximum DUST score of 20 or 30 (e.g. [12]). Pre-mapping DUST-filtering is sometimes done much more stringently (e.g. [6]), so that this is not an issue after mapping, but this strategy results in the loss of lower complexity reads mapping to genuinely ancient taxa, and may not be ideal for downstream population genetic or phylogenetic analyses. In bamdam compute, we calculate mean DUST score across all the reads assigned to a node or its descendants. We also provide a non-default option in bamdam shrink to filter out low-complexity reads, with a user-defined DUST threshold. The Prinseq documentation suggests that scores over 7 indicate low complexity [44].

Lastly, for ancient environmental DNA, a necessary diagnostic of ancientness is postmortem damage. Ancient taxa sequenced using single-stranded library prep methods show an accumulation of C-to-T transitions on both 5’ and 3’ ends of a read, whereas double-stranded methods show an accumulation of C-to-T on the 5’ and G-to-A on the 3’ end of the read. In a non-metagenomic context (eg in mapDamage [46]), at a given position in a read, the C-to-T frequency is the number of times we see a C-to-T transition over all reads, divided by the number of times we see a C-to-anything (including C-to-C) over all reads. For this, at a given position, we only consider reads in which the reference had a C at that position. In an ancient metagenomic context, we are interested in damage per taxonomic node instead of on single reference genomes, and a read may have multiple alignments to different references, so it is unclear how exactly to define those reads in which “the reference” had a C. There are several potentially reasonable ways to deal with this. One could (1) simply choose the best alignment per read, then average across all of the reads assigned to a taxonomic node or its descendants to obtain a damage estimate for that taxonomic node (bamdam mode 1, default). In the case when there are multiple best alignments with the same alignment score, bamdam chooses one of the best alignments randomly. One could (2) average all alignments per read to obtain a per-read proportion, then average the read proportions, weighting each read equally (mode 2). One could also (3) weight all alignments equally per taxonomic node, regardless of the number of alignments per read (mode 3). These three modes are implemented in bamdam. A detailed explanation and examples are given in the Additional file 1.

In non-metagenomic contexts (e.g. human aDNA), the normal approach is to keep one (best) alignment per read, then use software such as mapDamage to estimate damage [46]. For comparability with these approaches, we suggest that bamdam’s mode 1 (best alignment per read) is the most consistent. In modes 2 and 3, including lower-scoring alignments and/or biasing calculations towards reads with many alignments may increase bias from repeated regions of the genome, to which reads may align many times. Furthermore, if a read has multiple alignments, of which some have damage and others do not, which are otherwise equivalent, the alignment without damage will be the best alignment, since damage is a mismatch and the alignment score depends on the number of mismatches. Since we would generally expect a genuine damage signal to impact every alignment, we might want to conclude this read is not at all damaged, behavior which is only captured in mode 1. In any case, it is important to be aware of exactly what is being calculated when we speak of damage in a metagenomic context. See Section 2.2 and Fig. 4 for an investigation of the difference the mode can make on real data.

In addition to the tsv file containing authentication metrics, bamdam compute outputs a text file containing all damage-relevant substitutions for each taxonomic node to pipe into a plotting function, described below.

Both bamdam shrink and compute read bam files iteratively in conjunction with the lca file and take advantage of the read-sorting, so that bam files are never held in memory in their entirety. This iterative process means that RAM usage is fairly small.

*Bamdam combine* merges multiple tsv files from bamdam compute into an abundance matrix, optionally including mean 5’ damage, *k*-mer duplicity, DUST scores, etc., generating a single comprehensive file for authenticating a taxonomic profile across samples. Output files include read-weighted means across input samples and per-sample values.

*Bamdam plotdamage* uses the substitution file(s) from bamdam compute to produce single- or multi-sample deamination plots for any taxonomic node. This function can take multiple samples as input to create multi-sample damage plots (see the example in Fig. 1).

*Bamdam extract* extracts all alignments from a bam file that belong to reads that have been assigned to a specific taxonomic node(s) or below into an output bam file. This can be useful for downstream population genetic analyses or phylogenetic placement. The header of the output bam file is subset to only include references which appear in alignments of the output bam file reads. Users may optionally also include only alignments to the top reference, and/or only the best alignment per read, where ‘best’ is decided by the alignment score (AS tag) and ties are broken by picking one of the best alignments randomly.

*Bamdam plotbaminfo* generates single- or multi-sample plots for read length and mismatch distributions.

*Bamdam krona* generates XML files which can be passed to the KronaTools [35] command ktImportXML to obtain interactive, multi-sample Krona plots in html format, in which each taxa can be colored by its 5’ C-to-T frequency. An example is shown in Fig. 1 for a sample containing authentic ancient DNA and a negative control. A summary plot is also generated, and all samples can be included in a single webpage. Each plot is annotated per taxa with its 5’ C-to-T frequency, *k*-mer duplicity, mean DUST score, and mean read length. These plots can be a fast way to screen metagenomic libraries for authentic ancient DNA and an efficient and user-friendly way to explore and share data.

### Case study on real data

**Fig. 2:**
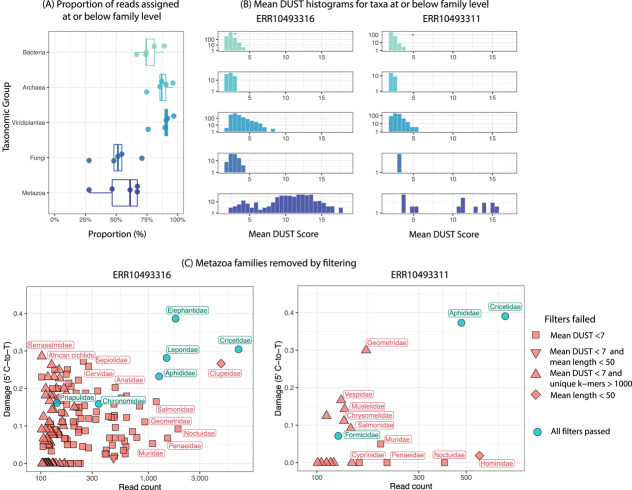
**A** The proportion of reads assigned at or below family level (e.g. genus, species, subspecies) for each of the taxonomic groups with the most reads assigned across 5 Kap Kopenhagen samples. These are the reads kept after bamdam shrink with default parameters. Each point here is a sample. **B** Mean DUST histograms for those taxa at or below family level with more than 100 reads, shown for the two largest samples. **C** The effect of filtering by DUST, mean read length, and unique *k*-mers on these two largest samples

As a case study, we used bamdam to explore five samples of various sizes from a recent shotgun sequencing ancient eDNA study of 2 million year old DNA from Northern Greenland [6]. We mapped reads against NCBI nt, RefSeq and PhyloNorway, similarly to the original study, ran ngsLCA [20], and then ran bamdam shrink and compute with default parameters in double-stranded mode (see Methods for details). Per-sample input bam sizes, peak RAM usage, clock time and file sizes for both bamdam shrink and bamdam compute are reported in Tables 1 and 2. Note that lca files have one read per row, so the file sizes are roughly proportional to the total number of reads before and after the shrink step, which by default removes all reads from the lca file and associated alignments for those reads from the bam file which do not fall at or under the family level. In this way, we can see that the majority of reads still remain after bamdam shrink. Figure 2A shows the proportion of reads which were assigned to taxonomic nodes at or below family level, i.e., those which were kept after bamdam shrink on default parameters. These proportions are shown across Bacteria, Archaea, Viridiplantae, Fungi and Metazoa, which together constitute the vast majority of the assignments. We can see that in this example, Archaea, Bacteria and Viridiplantae have reads which are assigned at taxonomic levels closer to the tips, on average, versus Fungi and Metazoa, and hence more of these reads are kept downstream. On the other hand, the reads that are removed are those making up the majority of the alignments, as reflected by the extent of bam compression in Table 1.

**Table 1:** File size comparisons for bamdam shrink

Sample	Input bam	Input lca	Output bam	Output lca	Bam Compression
ERR10493332	2.2 GB	31 MB	198 MB	24 MB	11.1$\times$
ERR10493362	25 GB	834 MB	1.8 GB	757 MB	13.9$\times$
ERR10493311	47 GB	2.0 GB	3.2 GB	1.8 GB	14.7$\times$
ERR10493315	49 GB	474 MB	1.4 GB	413 MB	35$\times$
ERR10493316	493 GB	25 GB	67 GB	23 GB	7.4$\times$

**Table 2:** Peak RAM and wall clock time for bamdam shrink and compute

Sample	Shrink peak RAM	Shrink time	Compute peak RAM	Compute time
ERR10493332	1.27 GB	3 min	1.08 GB	49 sec
ERR10493362	3.38 GB	31 min	5.21 GB	27 min
ERR10493311	3.33 GB	1 h 2 min	4.79 GB	26 min
ERR10493315	3.17 GB	56 min	3.62 GB	8 min
ERR10493316	19.79 GB	11 h 6 min	17.83 GB	8 h 6 min

**Fig. 3:**
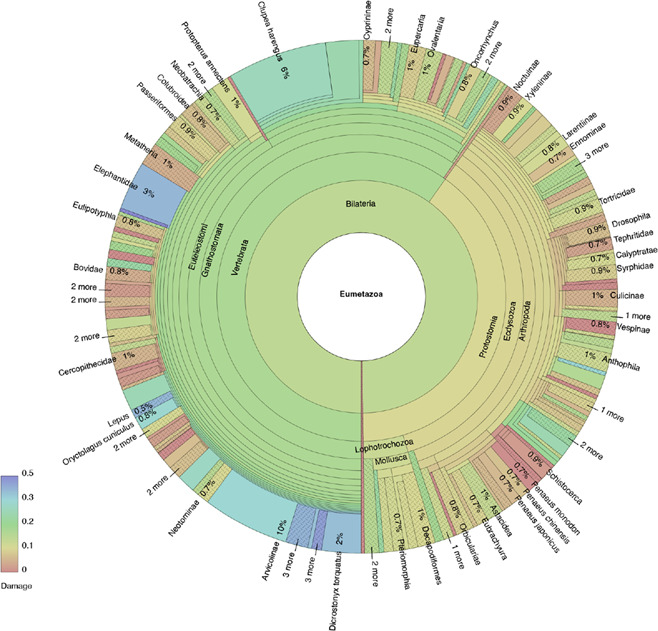
A screenshot of an (unfiltered) damage-coloured Krona plot for Metazoa, produced with bamdam and KronaTools [35] from Kap Kopenhagen sample ERR10493316. Wedge colour is by 5’ C-to-T frequency. An interactive version is available at https://github.com/bdesanctis/bamdam/examples/kapk_Metazoa.html (make sure to click “Colour by damage”). Within the interactive version, DUST values, *k*-mer duplicity, etc. can be seen per taxa by clicking on individual taxa

In Fig. 2B we plot DUST score distributions across all Metazoa families with more than 100 reads for the largest two samples. Note that the Prinseq documentation [44] suggest that DUST scores more than 7 should be considered “low complexity”. Based on this, we next chose to investigate Metazoa in more depth. We kept only reads in the bamdam compute output tsvs within Metazoa (with a bash grep command), and used bamdam krona and KronaTools ktImportXML function [35] to create damage-coloured Krona plots for Metazoa, available here https://bdesanctis.github.io/bamdam/example/kapk_subset_metazoa.html (make sure to click ‘Colour by damage’ on the left). A screenshot of the Krona plot for the largest sample is also shown in Fig. 3. Coloured in darkest blue, for example, we see the family Elephantidae, the genus *Lepus* and the subfamily Arvicolinae of Cricetidae, all reported in the original study. However, in this sample for Metazoa in particular, and similarly in the other samples included here (see Krona plots just linked), there remain many other damaged taxa which could potentially be considered authentic.

We imposed a filter across these two samples on Metazoa families with more than 100 reads requiring a mean DUST score of less than 7, a mean read length of less than 50 (a dataset-specific choice), and a unique *k*-mer count of more than 1000 (as in [12]). Bamdam uses $k=29$ by default. Figure 2C shows the effect of these filters. In this case, most taxa are removed by the DUST threshold, which indicates that filtering by DUST score is worthwhile. Across these two samples we keep multiple families which were identified in the original study, such as Cricetidae, Elephantidae, Leporidae, and Formicidae. We also see Aphididae kept in both samples with high damage and read counts, which was not identified in the original study. ERR10493316 contains damaged, low-complexity reads mapping to both Anatidae and Cervidae (mean DUST scores 9.6 and 13.0, respectively). These taxa were identified in the original study, but importantly, not in these samples. This is likely because the original study stringently DUST-filtered before mapping, and these identifications in other samples had sufficiently many high complexity reads assigned to them.

The tendency of read sets assigned to Metazoa families to have high DUST scores in this example reflects both the composition of these samples and the composition of the reference database used for mapping. We suspect that many of these Metazoa low-complexity identifications can be at least partially attributed to genuinely ancient sequences which do not have true representative sequences in our reference databases, and therefore spuriously map to a low-complexity region of a high-quality Metazoa reference genome. RefSeq is rich in high-quality Metazoa reference genomes. Indeed, many if not all of the families with high read count, high damage, and low complexity (that is, high mean DUST) filtered out in Fig. 2 have at least one chromosome-level assembly in RefSeq. Presumably genuine modern contaminants, such as Hominidae (bottom right of Fig 2C), are high complexity, but are filtered out by a mean read length threshold.

Finally we sought to investigate the impact of different computation strategies on damage. As mentioned earlier, because reads often have multiple alignments, bamdam compute offers three strategies with which to calculate substitution frequencies, damage, reference GC content, and divergence for each taxonomic node. All strategies aggregate across all reads assigned to that node or its descendants. The modes are discussed in more detail above and in Additional file 1, but briefly, the modes are: (1) use the best alignment per read, where “best” is determined by alignment score (the AS tag in a bam) and ties are broken by choosing a random best alignment, then weight each read equally, (2) average over all alignments per read, then weight each read equally, or (3) weight all alignments equally, regardless of how many alignments there are per read. Mode 1 is also the default in bamdam. Bamdam compute times and peak RAM usages for different modes are shown in Table 3. We did not run the largest sample on modes 2 and 3 because it was too slow. Mode 1 is substantially faster because it only has to parse one alignment per read.

**Table 3:** Peak RAM and wall clock time for compute modes

Sample	Mode 1 time	Mode 2 time	Mode 3 time	Mode 1 RAM	Mode 2 RAM	Mode 3 RAM
ERR10493332	49 sec	7 min	9 min	1.08 GB	1.21 GB	1.19 GB
ERR10493362	27 min	1 h 53 min	2 h 21 min	5.21 GB	5.48 GB	5.35 GB
ERR10493311	26 min	3 h 8 min	4 h 2 min	4.79 GB	5.04 GB	4.93 GB
ERR10493315	8 min	1 h 28 min	1 h 57 min	3.62 GB	3.88 GB	3.79 GB
ERR10493316	8 h 6 min	x	x	17.83 GB	x	x

**Fig. 4:**
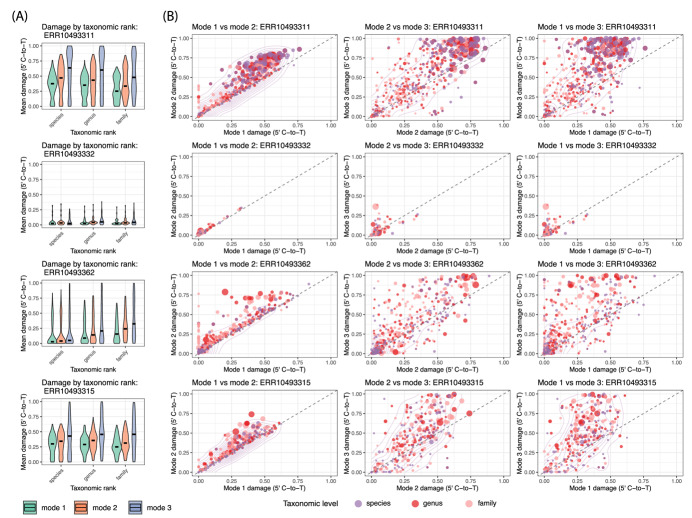
**A** A comparison of damage (5’ C-to-T) distribution violin plots across different computation modes in bamdam compute for four Kap Kopenhagen samples, by taxonomic rank, across those taxa with more than 100 assigned reads. **B** Direct comparison of mode 1, 2 and 3 in the same four samples. Each point is a taxa with more than 100 assigned reads (species, genus or family level). Dot sizes correspond to the mean number of alignments per read for that node (i.e. the total alignments divided by the total reads)

**Fig. 5:**
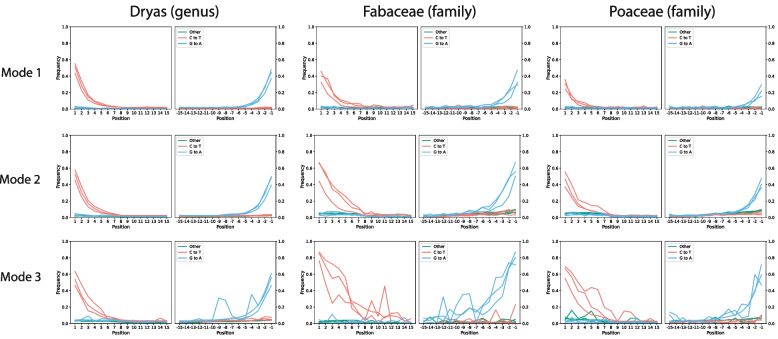
Multi-sample deamination plots for *Dryas*, Fabaceae and Poaceae for three Kap Kopenhagen samples. Each plot has 3 samples plotted on top of each other. In the samples ERR10493311, ERR10493315, and ERR10493362 respectively, *Dryas* had 2,533,043, 255,347, and 14,470 reads, Fabaceae had 1,026, 1,394 and 3,113 reads, and Poaceae had 82,523, 1,306, and 934 reads. Sample ERR10493332 had less than 100 reads assigned to each of these taxa and is not represented here

Figure 4A shows the difference in 5’ C-to-T frequency values across three modes for three taxonomic levels for four different samples, across all taxa with more than 100 reads. Mode 1 (use the best alignment only for each read, then average over reads) estimates the lowest mean damage, followed by mode 2 (average over alignments per read, then average over reads), and then mode 3 (average over alignments). Figure 4B are scatterplots where each point is a taxonomic node at the species, genus or family level with more than 100 reads. Modes 1 and 2 correlate reasonably well to each other, but mode 3 does not correlate particularly well to either mode 1 or 2. In both cases, taxa with less average alignments per read (where mean alignments per read within that taxa corresponds to dot size) produce slightly better correlations. Last, we wanted to know if the deamination plots themselves looked substantially different in other ways when the 5’ C-to-T frequency was different between modes. For this, we chose three taxa of interest that were present in multiple samples with high read counts: the genus *Dryas*, the family Fabaceae and the family Poaceae. We next used bamdam plotdamage to plot multi-sample deamination plots for these. In the samples ERR10493311, ERR10493315, and ERR10493362 respectively, *Dryas* had 2,533,043, 255,347, and 14,470 reads, Fabaceae had 1,026, 1,394 and 3,113 reads, and Poaceae had 82,523, 1,306, and 934 reads. One can see in Fig. 5 that, even with these high read counts, modes 1 and 2 produce much smoother deamination plots than mode 3.

## Discussion

Bamdam is a post-mapping toolkit for ancient environmental DNA, meant to be implemented after a lowest common ancestor assignment on a bam file. First, this software provides functionality to remove phylogenetically uninformative reads and alignments from a bam file, often reducing bam sizes by a factor of 10x or more, lowering storage needs for environmental DNA projects. Second, bamdam computes important authentication metrics such as mean read complexity (DUST scores), damage, and unique *k*-mers on every taxonomic node. Lastly, bamdam includes additional functions for downstream analysis and plotting. Bamdam processes large bam files in hours on a single thread, and uses little enough RAM that it can usually be run on a laptop computer if needed.

Of course, the metrics presented here will not capture all false positives in ancient metagenomic analyses that arise from all-vs-all alignment strategies, and considerable work remains in this space. Existing complementary approaches include the explicit modeling of per-taxa damage patterns [33] and pre-screening of reference databases for contamination [29]. Furthermore, many of the metrics in bamdam are also adapted from established methods developed in other contexts, building on tools and ideas that have already proven useful. For example, duplicity was first introduced in KrakenUniq [12], and some of the standard statistics included in bamdam such as mean read length, mismatch distributions and PMD scores are also available in aMeta, PMDTools, metaDMG, and other ancient DNA software [27, 33, 37]. Here we have integrated many of these concepts into a form tailored specifically for ancient metagenomic alignment and LCA data.

## Conclusion

The bamdam toolkit provides a critical set of authentication metrics and utilities for ancient metagenomic studies, addressing the pressing need for storage optimization and robust authentication in the field. By implementing *k*-mer duplicity, mean read complexity, and postmortem damage analysis in a lightweight and accessible format, bamdam empowers researchers to efficiently authenticate taxonomic assignments after the mapping and LCA steps. This comprehensive authentication approach is useful for reliable interpretation of ancient environmental DNA data, as it helps distinguish genuine ancient taxa from false positives caused by reference database contamination, lab artifacts, and mapping ambiguities. We hope that bamdam encourages ancient metagenomics researchers to explore and understand their own data.

## Methods

### Software

The bamdam toolkit is implemented in Python 3, and relies on pysam for bam parsing [47], hyperloglog for *k*-mer counting [48], tqdm for optional progress bars [49], and matplotlib for optional plotting [50]. It is compatible with Linux and macOS, and installable via source or pip. It is available at https://github.com/bdesanctis/bamdam/ under an MIT license.

### Performance on real data

Three raw fastq files from [6] were downloaded from the ENA project accession PRJEB55522. Adapter and quality trimming were performed using AdapterRemoval v2.3.4 [51] with the following options: –trimns and –trimqualities to remove ambiguous and low-quality bases, –mm 3 to allow up to three mismatches for adapter removal, –collapse to merge overlapping read pairs, –minalignmentlength 11, and –minlength 30. After concatenating all output reads, duplicate read names were removed using SeqKit v2.8.2 rename [52]. Further filtering was conducted using fastp v0.23.4 [53] with –dedup (duplicate removal), –dup_calc_accuracy 3, –low_complexity_filter with –complexity_threshold 30, –qualified_quality_phred 30 and –average_qual 25 (stringent quality thresholds), -l 30 (minimum length), and poly-G/X trimming (–trim_poly_g, –poly_g_min_len 6, –trim_poly_x, –poly_x_min_len 6). Reads were then complexity-filtered with SGA v0.10.15 with a DUST threshold of 30, and unique sequences were retained using VSEARCH v2.27.0 –fastx_uniques –minseqlength 30 –strand both [45, 54].

Similarly to the original study, these samples were mapped against NCBI nt (downloaded on July 31, 2023, and supplemented by blast build Oct 19, 2023), all of RefSeq (release 222 on January 8, 2024) and the Phylonorway database (from [7]), split into a total of 85 pieces, with bowtie2 version 2.5.4 and keeping up to 1000 alignments per read per database piece (-k 1000) [18]. Bam files were merged and query-sorted using samtools [32]. Lca files were made using a $95\%$ minimum similarity cutoff with ngsLCA v0.9 [20]. Various bamdam functions were used to make figures. Bamdam krona xml files were turned into html files using KronaTools ktImportXML function [35].

Peak RAM usage, clock time and file sizes for both bamdam shrink and bamdam compute were computed using the bash time module. This benchmarking was run on a cluster node of the UCSC Genomics Institute server, a system equipped with Intel Xeon X7560 CPUs at 2.27 GHz, and all tests were performed using a single thread.

## Supplementary Information

Additional file 1: Supplementary information discussing bamdam compute modes.

Additional file 2: Peer review history.

## Data Availability

Bamdam is open access and available under an MIT license on Github at https://github.com/bdesanctis/bamdam/ [55] and through Zenodo at https://doi.org/10.5281/zenodo.17642684 [56]. Data analyzed in the benchmarking section was published in Kjaer et al. 2022 [6], and raw files were downloaded from the ENA accession PRJEB55522 [57].
