# Supplementary material for: Bamdam: a post-mapping authentication toolkit for ancient metagenomics
Source: Genome Biol. 2025 Dec 5;26:413. doi: 10.1186/s13059-025-03879-x (PMC12679796; doi:10.1186/s13059-025-03879-x)
Supplement: Supplementary file 1 — Additional file 1: Supplementary information discussing bamdam compute modes. [file 13059_2025_3879_MOESM1_ESM.pdf]

Additional File 1 for bamdam: A post-mapping authentication toolkit for ancient metagenomics

1 Detailed explanation of compute modes

In this section we will give a detailed explanation and an example of the different modes implemented in bamdam compute. The mode is relevant for the computation of anything that differs across alignments for the same read; in bamdam, this is damage, reference GC content, ANI (average nucleotide identity), and the substitution file. Here, we focus first on damage.

Postmortem damage accumulates near the ends of reads in ancient DNA. The way this shows up in our data depends on the library prep method. In single stranded libraries, we see an accumulation of C-to-T transitions on the 5' and 3' ends of reads, and in double stranded libraries, an accumulation of C-to-T transitions on the 5' and G-to-A on the 3'. Without loss of generality, to simplify this discussion, we will refer only to C-to-T transitions in this section.

1.1 The non-metagenomic context.

First, let us discuss the postmortem damage calculation in a non-metagenomic context, in which we only keep one alignment for each read, and there is one reference genome. In this case, talking about mismatches in 'reads' and in 'alignments' is essentially synonymous. Here, we calculate the rate of C-to-T transitions across the ends of all of the reads that map to the reference genome for each position x. That is, we calculate for each position x

$$\text{C-to-T frequency} = \frac{\#(\text{reads with C-to-T})}{\#\ (\text{reads with C-to-anything})} \tag{1}$$

$$= \frac{\#\ \text{C-to-T}}{\#\ \text{C-to-T} + \#\ \text{C-to-G} + \#\ \text{C-to-A} + \#\ \text{C-to-C}} \tag{2}$$

That is, for the denominator we count all of the reads for which the reference base was a C, and for the numerator we count the subset of those reads for which the read base was a T. In Figure S1 here we show a deamination plot from mapDamage for a single-stranded library. The red line shows the C-to-T frequency across positions, and the blue line shows the G-to-A frequency across positions. The left side, with the positive numbers, is the position's distance into the 5' end, and the right side, with the negative numbers, is the position's distance into the 3' end. That is, position +1 (on the left side) refers to the first base on the 5' end of the reads. Since the plot goes +1 to +25 and -1 to -25, if a read only has 40 bases, the middle 10 of those bases will be represented both in [20,25] and [-20,-25].

Since frequencies are calculated for each position independently, for simplicity's sake, let us drop any mention of a specific read position x for the rest of this section and just refer to 'the C-to-T frequency'.

1.2 The metagenomic context

In the metagenomic lowest common ancestor context discussed in this paper, we keep multiple alignments per read, which may map to different references and/or different taxa. Each individual read is 'assigned' to the taxonomic node which is the lowest common ancestor of all the taxa it aligned to. This may not necessarily use all of the alignments - in ngsLCA, the lowest common ancestor can be chosen using only a

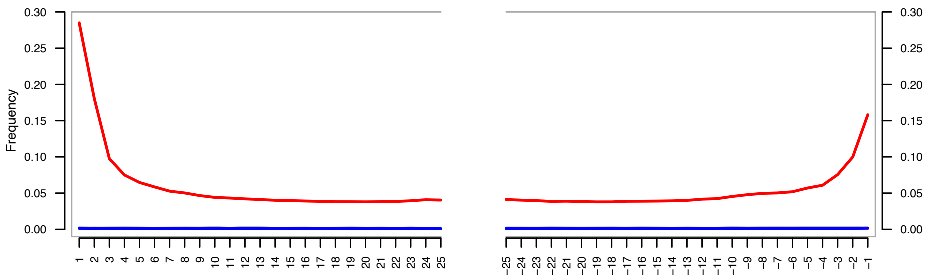

Fig S1: An example mapDamage plot for a single stranded library.

subset of alignments with a minimum similarity to their references. Then in bamdam, the same minimum similarity threshold can be used so that only this same subset of alignments is included in the shrunken bam file. In this way, only the alignments used to decide the LCA are carried forward. Bamdam computes statistics on each taxonomic node, using all of the reads assigned to that node or to its descendants, and (potentially) using all of the alignments which contributed to that read's assignment to that node. For example, information computed for a family-level node will be computed across all of the reads (and potentially all of their alignments) assigned to that family, or to any of the genera or species underneath it. On the other hand, information computed for a species-level node will never include those reads assigned to the genus above it, or any of their alignments. In this way, when we talk about 'all the reads' relevant for a node, we refer to all the reads assigned to it or its descendants. To be clear, here we are discussing how to deal with the fact that reads have multiple alignments in the context of these bamdam computations, *not* in the context of the lowest common ancestor step, which we are taking for granted. In particular, let us focus on how to compute C-to-T frequency for a taxonomic node when some or all of its relevant reads (assigned to that node or its descendants) have multiple alignments.

Different alignments of the same read may have different reference bases. When a read base is a T, some of its alignments may show a C-to-T, which would contribute to the both the numerator and the denominator of Equation 1. Other alignments of the same read, however, may show a G-to-T, A-to-T or T-to-T, which would contribute to neither. Similarly, when a read base is a C, some of its alignments may show a C-to-C, which would contribute to the denominator, but other alignments may show a G-to-C, which would not. Furthermore, some alignments may have higher scores than others, as determined by the alignment score, i.e. the AS tag in a bam file. In this context, it is not immediately clear how to calculate the numerator or the denominator in Equation 1. More generally it is unclear how to come up with the substitution matrix for each taxonomic node, which contains the frequency of not just C-to-Ts, but of every substitution for each position for each node, used for damage plotting. We offer a few options in bamdam. In all cases described here, it does not matter which reference genomes the individual alignments are to, since the LCA has already been decided.

Toy example. Let us first describe an example with which to illustrate each mode. Say we have two reads; the first with one alignment with a C-to-C, and the second with a thousand alignments: four hundred alignments with a C-to-T and another six hundred alignments with a T-to-T. In this example, assume also that the best alignment of the second read is one of the six hundred with a T-to-T; this is in fact the far more likely scenario, given that the alignment score depends on the number of mismatches.

Mode 1. Perhaps the easiest option conceptually is implemented as **mode 1** in bamdam: just use the one best alignment for each read, ignore the rest of the alignments altogether, and directly apply Equation 1. Here, the 'best' alignment is determined by the AS tag, and if there are multiple best alignments, one of them is chosen uniformly at random. We suggest that this approach in bamdam is the most comparable to the non-metagenomic context. This behavior is also similar to that of some pairwise aligners, e.g. if there are multiple best alignments, bowtie2 will report a random one by default [1]. In the toy example given, mode 1 would calculate the C-to-T frequency as 0, since there is one read with a C-to-C and no reads with a C-to-T (using their best alignments).

Mode 2. Say we want to take into account all of the alignments for each read, and to weight each read equally. To do this, we can create vectors for each read with the proportion of each possible reference-read base combination across all of its alignments, then average those vectors across reads to calculate the overall C-to-T frequency for the node. In particular, these vectors will contain entries for the proportion

of each of the 16 possible pairs of mismatches: C-to-C, C-to-T, C-to-G, C-to-A, G-to-G, G-to-A, etc. Let's return to the above example. The first read, which has one alignment with a C-to-C and no other alignments, will have a vector with a proportion of 1 for C-to-C and 0 otherwise. The second read, with four hundred alignments with a C-to-T and another six hundred with a T-to-T, will have in its vector a 400/1000 for C-to-T, a 600/1000 for T-to-T, and 0 otherwise. Averaging these vectors over the two reads then yields a proportion of 0.5 for C-to-C, a $(400/1000)/2 = 0.2$ for the C-to-T, and a $(600/1000)/2 = .3$ for T-to-T, and 0 otherwise. Note that T-to-T mismatches are not considered in the C-to-T frequency calculation as described above. The C-to-T frequency for this node is then calculated as

$$\text{C-to-T frequency} = \frac{\text{proportion C-to-T}}{\text{proportion C-to-anything}} = \frac{0.2}{0.5+0.2} = \frac{2}{7} \tag{3}$$

Mode 3. Both modes 1 and 2 are averaging over reads, so that each read contributes equally to the C-to-T frequency for the node. Another option, implemented as **mode 3** in bamdam, is to average over all of the alignments across all of the reads, so that each *alignment* instead contributes equally. That is, we ignore the read names entirely, make a list of all the alignments of all the reads assigned to a taxonomic node or its descendants, and calculate (for each position)

$$\text{C-to-T frequency} = \frac{\#(\text{alignments with C-to-T})}{\#\ (\text{alignments with C-to-anything})} \tag{4}$$

This will give 400/401 for the toy example scenario.

We can see a dramatic difference in the values computed in each mode for the toy example: mode 1 will give a C-to-T frequency of 0, mode 2 will give 2/7, and mode 3 will give 400/401.

Of course, there are still more options which are not implemented here. For example, one might only wish to conclude that a read has a C-to-T transition only if some minimum proportion of its alignments do. One might decide to include all alignments for each read, such as in mode 2, but downweight those with lower alignment scores. Or one might somehow take into account the specific reference genomes or taxa of each alignment, which we have not done at all here. In any case, it is important that we know exactly what is being calculated when we speak of damage in a metagenomic context.

1.3 Other statistics

In bamdam, the mode also affects the substitution file, the ANI, and the reference GC content.

The substitution file contains X-to-Y frequencies for each position in [+1,+15] and [-1,-15], where X and Y are in (A,C,T,G). These frequencies are calculated the exact same way as the C-to-T frequency, which was described above.

ANI (average nucleotide identity) uses the NM tag from the bam, the number of mismatches. In mode 1, ANI is calculated per read as (read length - NM) / read length using only the best alignment, then averaged over reads. In mode 2, ANI is also calculated per read, and we obtain the mean NM tag for a read over all its alignments, then put that in the equation, so it's (read length - meanNM) / read length, then finally we average over reads. In mode 3, we calculate ANI per alignment, as (read length - NM) / read length, then average that over all the alignments. Since the best alignment is usually the one with the least mismatches ('best' is based on the alignment score, which is lower with mismatches), mode 1 will estimate the highest ANI.

Reference GC content works the same way across modes. We use the MD tag and the cigar string to reconstruct the reference sequence for each alignment, or whenever needed. In mode 1 the ref GC content is calculated per read, using the best alignment, and then averaged over all reads. In mode 2 we find the average ref GC for a read across all of its alignments, then average over reads. In mode 3 we find the ref GC for each alignment, and average over alignments.

References

[1] Langmead B, Salzberg SL. Fast gapped-read alignment with Bowtie 2. Nature Methods. 2012 Mar;9(4):357–359. https://doi.org/10.1038/nmeth.1923.
