# Supplementary material for: Bamdam: a post-mapping authentication toolkit for ancient metagenomics
Source: Genome Biol. 2025 Dec 5;26:413. doi: 10.1186/s13059-025-03879-x (PMC12679796; doi:10.1186/s13059-025-03879-x)
Supplement: Supplementary file 2 — Additional file 2: Peer review history. [file 13059_2025_3879_MOESM2_ESM.docx]

Additional File 2: Peer Review History

**First round of review**

**Reviewer 1**

**Were you able to assess all statistics in the manuscript, including the appropriateness of statistical tests used?** Yes

**Comments to author:**

The authors propose a post-mapping authnetication tool, bamdam, for environmental ancient DNA analysis. The tool accepts a bam-file and a least common ancestor output from ngsLCA / sam2lca and performs shrinking of the bam-file as well as reports a number of authentication metrics including their visualization. The manuscript is clearly written and may be interesting for the ancient DNA community. However, I have major concerns about the novelty and impact of the present work. Below, I list my major and minor comments.

1) First of all, there are quite few other tools (e.g. mapDamage, PMDtools, MaltExtract, aMeta, HOPS, AMBER, metaDMG, DamageProfiler, Kraken2 / KrakenUniq etc.) that provide most if not all the authentication metrics mentioned in the manuscript in Figure 2. The authors mention many of them but do not do benchmark comparison of bamdam against them in order to demonstrate in what way bamdam is novel. The only obvious advantage of bamdam compared to the listed tools, that I understood from the manuscript, was that bamdam can shrink large bam-files in order to save disk space, which I do not think makes large impact for the computational analysis in this field.

2) Second, related to the shrinking bam-files, the purpose of LCA is in fact to retain multi-mapping reads in the analysis and not remove them. I understand the idea of the authors that reads assigned to a phylum or order might be less informative than e.g. species- and genus-level assignments for identification of a particular organism of interest. However, this is an opposite to LCA step and seems counter-intuitive: why to compute LCA in order to later shrink the bam-file, i.e. ignoring LCA results?

3) Third, the proposed tool in general seems to be taking one particular technical niche related to optimizing computational resources, which is, while technically interesting, lacks scientific impact. Although the authors demonstrate deep understanding of the subject, I do not see conceptually novel solutions in the proposed tool.

4) I do not think that disk space is a particularly acute problem for environmental ancient DNA research field. Environmental aDNA samples are typically not as deeply sequenced as samples used for population genomics analysis because genetic variation is typically not the major goal of environmental aDNA analysis where “presence-absence” of an organism is currently of major interest. Alternatively, in bioinformatics, for saving disk space one could for example convert bam-files to CRAM format, which would at least 2-3 times reduce disk space usage.

5) I do not understand the necessity to compute k-mer duplicity from a bam-file as proxy for evenness of coverage if one can simply compute the evenness of coverage from the bam-file with “samtools depth”. In addition, the k-mer duplicity seems to be a re-implementation of duplicity from KrakenUniq https://genomebiology.biomedcentral.com/articles/10.1186/s13059-018-1568-0, please see Figure 1. Is the k-mer duplicity different from the duplicity implemented in KrakenUniq?

6) The DUST scores described on page 5, lines 170-183, seem redundant to me as reference databases are dusted by default on their building step. This is, for example, a standard routine in Kraken family of tools.

Minor comments:

7) Are the authors sure that k-mer duplicity should be called “a proxy for evenness of coverage” and not “breadth of coverage”? The number of unique k-mers (or duplicity) does not provide information about their physical placement along the reference genome, therefore breadth of coverage seems to be a more correct definition.

8) Page 2, lines 54-55, the sentence about “limitation” of k-mer based methods because they “identify fewer taxa” should be re-phrased. It makes a wrong impression that a lot of detected taxa (even though many of them can be false-positive) can be considered as a criterion of successful analysis.

9) Figure 2, please use a, b, c, d instead of “top row” and “bottom row”

10) Page 7, line 249, here and in a few other places it is mentioned that the goal of shrinking bam-files is to remove “uninformative reads”. I would be careful to cal these reads “uninformative” as they were retained by LCA on purpose. Otherwise, aligning with bowtie2 and ignoring multi-mappers would suffice as well.

11) Out of curiosity, why Penaeus and Coregonus are called “common database contaminants” on page 7, line 242? Was it reported in any study, any reference? Can the authors specify what databases contain these typical contaminants?

12) In my opinion, it makes a lot of sense to include bamdam as an additional technical module for ngsLCA+metaDMG (Holi pipeline) rather than present bamdam a separate tool.

**Reviewer 2**

**Were you able to assess all statistics in the manuscript, including the appropriateness of statistical tests used?** Yes

**Comments to author:**

De Sanctis et al. developed a new suite of SAM/BAM related Python scripts that aim to increase the effiency in processing sequencing data that were aligned to large reference databases using BWA or BowTie2 and have least-common-ancestor assignment information. Their scripts can be largely divided into two set of commands. The first one is to reduce the size of the obtained alignment files by discarded sequences that do not fulfil user-specified criteria ("bamdam shrink"). The second one is to commute authentication statistics, such as the amount of ancient DNA damage present in the samples or a k-mer based heuristic for the eveness of coverage ("bamdam compute"). With their program "bamdam", the authors aim to fill the gap that there is currently no post-processing suite for environmental metagenomic DNA that is accessible for researchers that do not have a strong bioinformatics background.

I think the authors indeed provide a set of tools that make a lot of common processing tasks easily available. The shrinking command elegantly deals with the issue that many alignments are uninformative and do not need to be kept. This reduces disk space and, in the long run, the environmental footprint of the analyses - an important consideration in today's attempts to reduce the emission of carbondyoxide. The computed authentication metrics are state of the art and the authors put additional thought in providing additional metrics, such as evaluating the evenness of coverage via k-mer duplicity or the DUST score of taxonomic nodes to flag potential sources of contamination. However, I have a few comments that I think could help to improve the manuscript and the tool suite.

Comments:

- line 67-69: "One can cut the reference database into smaller pieces, index and map separately, and merge bam files afterwards without too much RAM being required in any one step." While this is technically true, this statement is misleading for the novice reader. The authors should mention here that MALT includes the LCA step directly while for BowTie2 and BWA the LCA step is run afterwards and therefore splitting the database is easily feasible. For MALT one should also be able to do the splitting if one would merge the resulting RMA6 files and run the LCA algorithm with MEGAN afterwards.

- line 125-126: "Next, bamdam compute processes the bam and lca files iteratively to output a tsv and substitution text file." The authors should clarify here and throughout the manuscript that the second file contains nucleotide substitutions as this is not apparent without inferring it from the use of the substitution text file in Figure 1.

- line 134-136: "All metrics weight each read equally, rather than each alignment, so that per-read statistics accumulate while alignments for the same read are being processed (...)" This sentence is not really clear. The authors should clarify how the statistics calculate across multiple alignments of the same read influence the reported metrics.

- line 139-141: "Many false positive taxonomic assignments involve reads which align only to one small region of a reference genome(s), rather than being approximately uniformly distributed." Either this should refer to a published figure or have a small illustration showing an examples.

- line 142-143: "However, evenness of coverage is calculated on a single reference genome." I don't think this statement is true. Either specify in which program this is calculated across a single reference genome or add "usually" to the sentence.

- line 168-169: "Depending on the dataset, a reasonable threshold may be to flag taxa with a duplicity above 1.5." I like this idea of thought in the previous lines and am therefore curious how the authors came to this conclusion. Is this based on empirical data? If yes, which one? This should also be stated for the statement in line 181-182.

- line 191-192: "This is calculated in a reference-free way by parsing the sequence, the MD tag and the cigar string." When a BAM file contains a MD tag, the information to reconstruct the reference from the sequence are stored for each read in this tag. However, for the calculation of the MD tag the reference genomes were necessary. I would therefore not classify this approach as reference-free as reference-free usually refers to k-mer based methods that do not include an alignment step.

- line 226-230: For reproducibility, it would be nice to have further information, e.g. the accession codes of the sequencing data, the accession date of the databases, and the versions of the software. Furthermore, if the software were run with non-default options, it should be stated, too.

- line 241-244: I would suggest to show the numbers either in a table or in a simple bar plot that additionally has the suggested thresholds for the DUST score and k-mer duplicity instead of the text. I think this would allow for an easier comparison of the values across the three taxa.

- discussion: in the introduction, the authors spent quite some time explaining while available programs that allow to align sequencing data and authenticate true alignments, such as aMeta, have short comings due to their alignment method, MALT. However, the authors lack to discuss which of the steps that they implemented into bamdam are also present in aMeta, which ones are only present in bamdam, which ones are only present in aMeta. I think the readers of this manuscript would highly benefit from such an overview.

Here are some general comments regarding the code provided on GitHub:

- Code installation: currently, there are only very rudimentary installation instructions available on the GitHub page. The instructions are based on the Python package index PyPI and use "pip" to install these but the authors do not provide any information on which Python version is required to be installed. Furthermore, the repository has to be cloned manually and the rights of the major Python script have to be changed to make it an executable. This is not really novice user friendly. Instead, I would highly recommend to convert the script into a conda package to increase the usability and allow a single-line installation of the software.

- Programming style: the whole suite of "bamdam" that consists of five main sub-commands is spread across 27 functions in a single file. The script comprises almost 1,500 lines of Python code and is very hard to review. I would suggest to modularise the code and split it into multiple files to increase the readability. Furthermore, adding some test cases that a user could run to test the software without requiring them to first run BWA/BowTie2 and then ngsLCA would be appreciated.

**Authors Response**

**Point-by-point responses to the reviewers’ comments:**

**Reviewer 1**

Author replies are in blue.

We greatly appreciate the comments from two reviewers and the guest editors of Genome Bi-

ology, and are grateful for the accepted appeal and the chance to respond to these comments. Our

responses are in blue. By incorporating these comments, we think both the manuscript and the

program have improved greatly since the last version.

We have now added two new modules to bamdam since its first submission, both which operate

on the tsv output from bamdam’s compute step. The first, called “combine”, is a way to combine

compute tsv files from multiple samples into a single abundance matrix, which can also optionally

contain mean damage, mean duplicity, etc. We expect this to be useful for downstream analysis

in cases where multiple samples need to be combined. The second, called “krona”, generates XML

files which can then be passed into the existing software KronaTools Ondov2011 to make html files

which are interactive ways to view the key features of ancient metagenomic experiments. In these

plots, we annotate for each taxa the damage (5’ C-to-T misincorporation frequency), duplicity,

mean dust score, and mean read length, and allow the option to colour taxa by their 5’ damage

values. A single html file can be generated for an entire ancient metagenomics experiment. We

have added a static example as part of the workflow and a link to an interactive version showcasing

this new function on real data on the github. Adding the ability to colour taxa in Krona plots by

damage yields an accessible and efficient way to explore ancient metagenomics data, and to quickly

tell apart modern from ancient taxa, which will in particular benefit those members of the ancient

environmental DNA community who are not bioinformaticians such as geologists or archaeologists.

Lastly, we extended the plotdamage (and plotbaminfo) function to take in multiple samples to

make multi-sample damage plots.

We have also rewritten a large proportion of the manuscript for clarity. In particular, the re-

viewer and guest editor comments made clear to us that we had not sufficiently communicated the

intended role of bamdam in the ecosystem of existing tools, which we have now clarified. We have

also added a new figure demonstrating the effectiveness of read set complexity as a novel way to

detect false positive taxonomic assignments in real data.

If there is a further round of reviews following these revisions, we would enthusiastically en-

courage interested reviewers to run the bamdam tutorial, which takes less than 20 minutes, does

not require your own data, and can be done on a laptop. The tutorial is available on the github

<https://github.com/bdesanctis/bamdam>.

**Guest editor comments**

In addition to addressing the reviewer comments, one of the guest editors feels the bench-

mark has fallen short of actually being a benchmark in that the manuscript does not include any

demonstration file size and memory requirements improve significantly compared to relevant tools.

They have suggested comparison be made at least to aMeta, HOPS, KrakenUniq and genozip.

As improved computational requirements is a key advance of bamdam this would be critical to

demonstrate an advance.

We appreciate these suggestions and agree that our testing on real data could be expanded, and

have done so (see below). From this comment especially, as mentioned above, we also now realize

we may not have fully communicated the role of bamdam in its intended pipeline. In particular,

bamdam itself is not doing read alignment or taxonomic profiling, the step that consumes by far the

most computing resources in aMeta, HOPS, KrakenUniq, and other taxonomic profiling software.

It is therefore not really comparable to these methods. Instead, as now stated in the title, the

abstract, and as shown in the workflow figure, bamdam is intended as a post-mapping, post-least-

common-ancestor module. The scope of bamdam is similar to that of mapDamage, DamageProfiler,

pyDamage or metaDMG. These tools, especially mapDamage, have become a standard in ancient

DNA and are critical for rigorous authentication by quantifying damage patterns. Similarly, bam-

dam provides analogous (and increased) rigor, but tailored to ancient metagenomics.

In theory we could compare sizes and runtime between bamdam to aMeta or HOPS by choosing

a pairwise aligner (e.g. bowtie2 or bwa), and comparing the runtime and file sizes in the aligner +

ngsLCA + bamdam pipeline to aMeta or HOPS. But this would essentially amount to comparing

the aligner choices – not bamdam – as this alignment step is by far the most computationally

intensive step in any of these pipelines, and comparing pairwise aligners such as bowtie2 vs bwa vs

HOPS etc is not in the scope of the current paper. Similarly, for example, one could not compare

reasonably runtime and file sizes between mapDamage to bwa. We did attempt to run aMeta on

the same samples used to demonstrate bamdam, as we would have liked to compare taxonomic

profiles, but doing this required more than 1.2Tb of RAM for this data and proved unfeasible on

our computing infrastructure.

Genozip is potentially the only suggested tool which might be directly comparable to bam-

dam’s functionality, in particular the bamdam shrink program, in terms of file size and memory

requirements. In this comparison, which we did test, bamdam outperforms Genozip substantially.

However, we did not add this comparison into the paper because we feel this is a misrepresentation

of bamdam’s capabilities; bamdam is not doing proper lossless compression as Genozip does. In

other words, bamdam “shrink” is lossy – even though the information lost is not needed – while

genozip (and other lossless compression methods) is not. We may have now clarified this in the

manuscript (line 141-142). Genozip is also not open source.

We appreciate and agree with the suggestion to expand the benchmarking section. We have

expanded the manuscript to include a demonstration of bamdam on a recently published ancient

metagenomics dataset, 2 million year old environmental DNA from Greenland. This section also

now includes a demonstration of the effectiveness of read set complexity as a novel authentication

metric. We believe this section will be valuable for the audiences of this paper since this offers a

direct example on the many new features that bamdam provides.

**Comments to author:**

The authors propose a post-mapping authnetication tool, bamdam, for environmental ancient DNA analysis. The tool accepts a bam-file and a least common ancestor output from ngsLCA / sam2lca and performs shrinking of the bam-file as well as reports a number of authentication metrics including their visualization. The manuscript is clearly written and may be interesting for the ancient DNA community. However, I have major concerns about the novelty and impact of the present work. Below, I list my major and minor comments.

1) First of all, there are quite few other tools (e.g. mapDamage, PMDtools, MaltExtract, aMeta, HOPS, AMBER, metaDMG, DamageProfiler, Kraken2 / KrakenUniq etc.) that provide most if not all the authentication metrics mentioned in the manuscript in Figure 2. The authors mention many of them but do not do benchmark comparison of bamdam against them in order to demonstrate in what way bamdam is novel. The only obvious advantage of bamdam compared to the listed tools, that I understood from the manuscript, was that bamdam can shrink large bam-files in order to save disk space, which I do not think makes large impact for the computational analysis in this field.

We appreciate these comments. We have covered the majority of these in our responses above

to the editor and guest editors, and in our appeal. Here we offer further information that we hope

will be useful for appreciating the unique features bamdam offers.

1. The shrinking of file sizes is indeed important. It will (i) speed up and reduce the computing

resource requirement for the remaining modules and all subsequent analysis; (ii) help resolve a

major block in metagenomics of data sharing and archiving (metagenomic alignment files are often

not shared and archived publicly due to their giant file sizes, despite bams commonly being re-

leased with other genomic studies); (iii) become more significant along with the rapid reduction in

sequencing costs (leading to even bigger metagenomic datasets) and increased amount of reference

genomes (such as DToL, B10K, etc. We now elaborate on these points in the manuscript (line

129-143).

2. While a few metrics do exist across various tools the reviewer mentioned, running many sep-

arate pieces of software — each with differing assumptions, taxonomic identification methods and

reference databases, and therefore using different taxonomic profiles and read sets — is not only

computationally prohibitive but also fundamentally incompatible for integrated analyses. More im-

portantly, however, none of these tools are designed for processing post-alignment files for ancient

metagenomic data that bamdam is specifically developed for, except metaDMG which generates

one matrix to access the DNA damage across nodes of taxonomic profile – a function also available

in bamdam. We will briefly address the tools listed. First, mapDamage models the DNA damage

of a individual taxon, as does DamageProfiler, AMBER assesses reference bias from a bam, and

PMDtools annotates individual reads in bam files with damage scores – but all of these are built

for single-taxa studies, not metagenomics, though otherwise comparable to bamdam in their in-

tended point in a workflow. Next, MALT relies on Megan, HOPS is built for pathogens relying on

MALT, MaltExtract summarizes MALT output, and aMeta integrates MALT and other tools into a Snakemake workflow. Critically, all of these tools rely on Megan through MALT, which has previously been shown to underperform when compared to ngsLCA. aMeta has the option to run bowtie2 and the single-taxa mapDamage instead of MALT, but does not include any further authentication options after this point, which is the main point of bamdam (functionality after taxonomic profiling). Indeed, one potential use for bamdam is after aMeta’s final bowtie2 step, which we have clarified in lines 90-92. Lastly, Kraken and KrakenUniq do not output alignments, which are strictly necessary for ancient DNA.

As stated in the introduction, ”There are no published methods which compute (these)

authentication metrics on a bam file with associated least-common-ancestor information”, which

is quickly becoming the favored route for taxonomic identification in ancient eukaryotic metage-

nomics. Indeed a recent paper published in Genome Biology stated that “bowtie2 + sam2lca should

entirely replace MALT while delivering alignments in bioinformatics-friendly SAM-format” ameta,

and bamdam is the first software which can compute these metrics on this format. We have added

to this to the manuscript (lines 81-83). Bamdam also introduces a novel authentication metric,

read set complexity, which we now demonstrate is an important filter for false positive taxonomic

identifications. Lastly, bamdam now offers damage-coloured Krona plots, making ancient metage-

nomic data more accessible to non-bioinformaticians such as ecologists, archaeologists or geologists,

all of whom frequently collaborate on ancient environmental DNA studies.

2) Second, related to the shrinking bam-files, the purpose of LCA is in fact to retain multi-mapping reads in the analysis and not remove them. I understand the idea of the authors that reads assigned to a phylum or order might be less informative than e.g. species- and genus-level assignments for identification of a particular organism of interest. However, this is an opposite to LCA step and seems counter-intuitive: why to compute LCA in order to later shrink the bam-file, i.e. ignoring LCA results?

The purpose of the LCA is to assign reads to taxonomic nodes on a taxonomy tree from read

alignments. In reality, a large number of reads originate from conserved genomic regions that are

aligned to many different taxa across the tree of life, thus assigned to a node placed high on the

tree (such as phylum) or even to the root of the tree of life if they map across kingdoms. These

reads have little or no taxonomic resolution thus have no ecological or biological meaning. Further-

more reads with more alignments are more likely to be assigned to levels higher up the taxonomy,

thus accounting for a large proportion of the file size. Effectively, this function exists as a filter

removing less phylogenetically informative reads; users can, of course, adjust the parameters for

applying this threshold as required for their own research needs. As reviewer 2 notes, “The shrink-

ing command elegantly deals with the issue that many alignments are uninformative and do not

need to be kept. This reduces disk space and, in the long run, the environmental footprint of the

analyses - an important consideration in today’s attempts to reduce the emission of carbon dioxide.”

3) Third, the proposed tool in general seems to be taking one particular technical niche related to optimizing computational resources, which is, while technically interesting, lacks scientific impact. Although the authors demonstrate deep understanding of the subject, I do not see conceptually novel solutions in the proposed tool.

This was also addressed in our appeal and above. Breifly: Bamdam’s shrink function is indeed

useful for data storage, archiving, and downstream interaction with bam files, but there are several

more important novel advances here. To our knowledge, there are no published methods yet which

compute authentication metrics on a bam file with associated least-common-ancestor information.

Even the aMeta paper, also published in Genome Biology, acknowledged this as a necessary next

step in ancient metagenomics (see above). One preprint, metaDMG, describes a software which

computes postmortem damage, but bamdam is an entire integrated toolkit and goes much further

than this. We introduce a new authentication metric for ancient metagenomics, read set complexity,

and demonstrate its importance in determining false-positive taxonomic identifications in Figure 3.

Stringent authentication is extremely important in ancient metagenomics, and has been the focus

of multiple recent papers (e.g. Oskolkov2025,Pach2024). Bamdam now includes many user-friendly

visualization tools, such as damage-colored krona plots and multi-sample damage plots. We ex-

pect this to be particularly useful for those working in, or collaborating on, sedimentary ancient

DNA projects but who are not themselves bioinformaticians or programmers; for example, there

are many ecologists, archaeologists, geologists and paleontologists in our field who currently find it

difficult to interact with and explore ancient metagenomic data.

4) I do not think that disk space is a particularly acute problem for environmental ancient DNA research field. Environmental aDNA samples are typically not as deeply sequenced as samples used for population genomics analysis because genetic variation is typically not the major goal of environmental aDNA analysis where “presence-absence” of an organism is currently of major interest. Alternatively, in bioinformatics, for saving disk space one could for example convert bam-files to CRAM format, which would at least 2-3 times reduce disk space usage.

Thank you for your thoughts. This specific point was discussed in our appeal and above. Con-

verting to CRAM format is not possible because this is a reference-based format, and metagenomic

reference databases can be many terabytes in size (e.g. RefSeq).

5) I do not understand the necessity to compute k-mer duplicity from a bam-file as proxy for evenness of coverage if one can simply compute the evenness of coverage from the bam-file with “samtools depth”. In addition, the k-mer duplicity seems to be a re-implementation of duplicity from KrakenUniq https://genomebiology.biomedcentral.com/articles/10.1186/s13059-018-1568-0, please see Figure 1. Is the k-mer duplicity different from the duplicity implemented in KrakenUniq?

Thank you for your suggestion. We have now clarified this in the manuscript (lines 177-179).

The k-mer duplicity is indeed the same as the one implemented in KrakenUniq - we were inspired by

it, and have now clarified this (lines 181-183). KrakenUniq, however, doesn’t produce alignments,

so doesn’t allow for the calculation of postmortem damage and is therefore incompatible with the

authentication of ancient environmental DNA. The ”samtools depth” on the extracted bam file is

a solution for one taxa at a time with one reference genome, and it is entirely intractible to look

at these graphs for every reference genome and taxa in every sample of a metagenomics experiment.

6) The DUST scores described on page 5, lines 170-183, seem redundant to me as reference databases are dusted by default on their building step. This is, for example, a standard routine in Kraken family of tools.

The reviewer is correct that dusted reference databases are commonly used in k-mer based

Kraken family tools, and have clarified lines (lines 204-205) but this is not typically the case in

this context; i.e. for whole read alignment based methods that require indexing reference genomes.

NCBI reference genomes, for example, are not dusted by default. In any case, a standard DUST

mask is around 20-30. For example, the database decontamination procedure in BLAST

(Camacho2009) uses NCBI’s Dustmasker, which has a default DUST mask of 20. However, the origi-

nal DUST algorithm defines “complex” as a DUST score less than 7. We find that, when a read set

for a taxonomic node has a DUST score between 10 and 20, it is usually a database contaminant.

We have now added a new figure illustrating this and the use of mean DUST scores on real data.

We have also clarified this in the manuscript (lines 195-210).

Minor comments:

7) Are the authors sure that k-mer duplicity should be called “a proxy for evenness of coverage” and not “breadth of coverage”? The number of unique k-mers (or duplicity) does not provide information about their physical placement along the reference genome, therefore breadth of coverage seems to be a more correct definition.

We appreciate the suggestion. We agree and have changed this throughout.

8) Page 2, lines 54-55, the sentence about “limitation” of k-mer based methods because they “identify fewer taxa” should be re-phrased. It makes a wrong impression that a lot of detected taxa (even though many of them can be false-positive) can be considered as a criterion of successful analysis.

Thank you for this comment. We agree and have removed this phrase.

9) Figure 2, please use a, b, c, d instead of “top row” and “bottom row”

Figure 2 has been significantly changed.

10) Page 7, line 249, here and in a few other places it is mentioned that the goal of shrinking bam-files is to remove “uninformative reads”. I would be careful to cal these reads “uninformative” as they were retained by LCA on purpose. Otherwise, aligning with bowtie2 and ignoring multi-mappers would suffice as well.

We appreciate this suggestion and the request for clarity. We have changed it to “phylogenet-

ically uninformative”. Ignoring multi-mappers would not suffice, because it would mean indexing

and mapping against a multi-terabyte database in a single chunk instead of in a few pieces and

later merging, which is computationally infeasible. Also, it would lead to decreased accuracy (see

ngsLCA paper).

11) Out of curiosity, why Penaeus and Coregonus are called “common database contaminants” on page 7, line 242? Was it reported in any study, any reference? Can the authors specify what databases contain these typical contaminants?

We appreciate these seem rather arbitrary in hindsight. These contaminants are in NCBI’s nt, a

commonly used database for BLAST and ancient eukaryotic metagenomics pipelines. We now provide a figure showing that Penaeus, among others, has an elevated DUST score in a real dataset.

12) In my opinion, it makes a lot of sense to include bamdam as an additional technical module for ngsLCA+metaDMG (Holi pipeline) rather than present bamdam a separate tool.

Yes, usage after ngsLCA (with or without metaDMG) is absolutely the context here, and we

should have emphasized this more. We have now rephrased the relevant parts the manuscript to

reflect this (lines 90-95).

**Reviewer 2**

**Were you able to assess all statistics in the manuscript, including the appropriateness of statistical tests used?** Yes

**Comments to author:**

De Sanctis et al. developed a new suite of SAM/BAM related Python scripts that aim to increase the effiency in processing sequencing data that were aligned to large reference databases using BWA or BowTie2 and have least-common-ancestor assignment information. Their scripts can be largely divided into two set of commands. The first one is to reduce the size of the obtained alignment files by discarded sequences that do not fulfil user-specified criteria ("bamdam shrink"). The second one is to commute authentication statistics, such as the amount of ancient DNA damage present in the samples or a k-mer based heuristic for the eveness of coverage ("bamdam compute"). With their program "bamdam", the authors aim to fill the gap that there is currently no post-processing suite for environmental metagenomic DNA that is accessible for researchers that do not have a strong bioinformatics background.

I think the authors indeed provide a set of tools that make a lot of common processing tasks easily available. The shrinking command elegantly deals with the issue that many alignments are uninformative and do not need to be kept. This reduces disk space and, in the long run, the environmental footprint of the analyses - an important consideration in today's attempts to reduce the emission of carbondyoxide. The computed authentication metrics are state of the art and the authors put additional thought in providing additional metrics, such as evaluating the evenness of coverage via k-mer duplicity or the DUST score of taxonomic nodes to flag potential sources of contamination. However, I have a few comments that I think could help to improve the manuscript and the tool suite.

We very much appreciated the assessment that bamdam fills a gap in the available software for

metagenomic analysis, and we have sought to make the post-mapping authentication options as

comprehensive and user-friendly as possible. We thank this reviewer for their comments on this.

Comments:

- line 67-69: "One can cut the reference database into smaller pieces, index and map separately, and merge bam files afterwards without too much RAM being required in any one step." While this is technically true, this statement is misleading for the novice reader. The authors should mention here that MALT includes the LCA step directly while for BowTie2 and BWA the LCA step is run afterwards and therefore splitting the database is easily feasible. For MALT one should also be able to do the splitting if one would merge the resulting RMA6 files and run the LCA algorithm with MEGAN afterwards.

Thanks for pointing this out. We agree and have added to the section describing MALT: ‘One

workaround is to cut the reference database into smaller pieces, map separately, and merge bam

files afterwards before running the MEGAN lca step.’ (lines 69-70).

- line 125-126: "Next, bamdam compute processes the bam and lca files iteratively to output a tsv and substitution text file." The authors should clarify here and throughout the manuscript that the second file contains nucleotide substitutions as this is not apparent without inferring it from the use of the substitution text file in Figure 1.

Thanks for this suggestion. We have clarified the Figure and this section, the latter of which

now says:

“Bamdam compute processes the bam and lca files iteratively to output two files: a tsv con-

taining authentication metrics and a nucleotide substitution frequency text file. Both output files

have one row per taxonomic node. The tsv file contains all of the authentication metrics and is

ordered by total assigned reads, and the substitution text file stores a list of nucleotide substitution

frequencies for each taxonomic node for damage plotting.”

- line 134-136: "All metrics weight each read equally, rather than each alignment, so that per-read statistics accumulate while alignments for the same read are being processed (...)" This sentence is not really clear. The authors should clarify how the statistics calculate across multiple alignments of the same read influence the reported metrics.

We have elaborated on and clarified this, and have moved the discussion to the postmortem

damage section in lines 219-231, which now reads:

“Importantly, this calculation is performed in bamdam such that each read is weighted equally,

rather than each alignment. In similar programs such as mapDamage or PMDTools mapdam-

age,pmdtools, postmortem damage frequency is calculated by averaging over all alignments. This

is acceptable in a single-source context, where it is common to only keep the best alignment for each

read. In a metagenomics context, however, one read may have thousands of alignments whereas

another may only have one or two, so that weighting each alignment equally may heavily bias

the postmortem damage towards reads with more alignments. Of course, for a given read, each

alignment may not have the same substitution profile. Here, the substitution profile for a given

read is averaged over all of its alignments, then each read is counted equally in the calculation of

postmortem damage. There are other reasonable ways in which this could be done - for example,

one could consider a position to carry a signature of postmortem damage only if every one of its

alignments contains that transition. Here we chose to average over alignments for each read so that

frequencies could reasonably be compared to baseline divergence, also calculated by weighting each

read equally.”

Like the reviewer, we were also interested in exactly how calculating these metrics across mul-

tiple alignments of the same read might bias postmortem damage estimates, but chose not to

speculate on it here because the issue appears to be more complicated than one might expect. We

began to test this on one dataset, and were surprised that the direction of the bias introduced by

calculating across alignments depends on the taxonomic level, moving from an underestimate to an overestimate with taxonomic depth – but never biasing postmortem damage on the 5’ end by

more than 0.05 in either direction. We feel that a full discussion of this, and indeed of the many

ways to calculate damage in a metagenomic context, merits a deeper investigation outside of the

scope of this paper.

- line 139-141: "Many false positive taxonomic assignments involve reads which align only to one small region of a reference genome(s), rather than being approximately uniformly distributed." Either this should refer to a published figure or have a small illustration showing an examples.

We appreciate the suggestion. This has been previously shown, e.g. see Figure 1 in Steineg-

ger2020. This note and reference has also been added to the manuscript (lines 169-171).

- line 142-143: "However, evenness of coverage is calculated on a single reference genome." I don't think this statement is true. Either specify in which program this is calculated across a single reference genome or add "usually" to the sentence.

We agree and have added “usually”.

- line 168-169: "Depending on the dataset, a reasonable threshold may be to flag taxa with a duplicity above 1.5." I like this idea of thought in the previous lines and am therefore curious how the authors came to this conclusion. Is this based on empirical data? If yes, which one? This should also be stated for the statement in line 181-182.

Thank you, we also like the idea of thought in the previous lines. We have tested more (un-

published) datasets since writing this and have decided to remove the suggested threshold in both

cases, since we have found that it depends strongly on the dataset. For line 181-182, we found a

reference and changed the suggestion to simply state: “For example, the Prinseq documentation

suggests that scores over 7 can be considered low complexity (Schmieder 2011).”

- line 191-192: "This is calculated in a reference-free way by parsing the sequence, the MD tag and the cigar string." When a BAM file contains a MD tag, the information to reconstruct the reference from the sequence are stored for each read in this tag. However, for the calculation of the MD tag the reference genomes were necessary. I would therefore not classify this approach as reference-free as reference-free usually refers to k-mer based methods that do not include an alignment step.

Yes, we agree and have changed this.

- line 226-230: For reproducibility, it would be nice to have further information, e.g. the accession codes of the sequencing data, the accession date of the databases, and the versions of the software. Furthermore, if the software were run with non-default options, it should be stated, too.

Thanks for this suggestion. We have added software versions, stated any non-default parame-

ters used, added database versions, and given accession codes for the sequencing data used.

- line 241-244: I would suggest to show the numbers either in a table or in a simple bar plot that additionally has the suggested thresholds for the DUST score and k-mer duplicity instead of the text. I think this would allow for an easier comparison of the values across the three taxa.

We completely agree. We have now added a new figure showing DUST scores, duplicity, and

damage for several samples from a published dataset.

- discussion: in the introduction, the authors spent quite some time explaining while available programs that allow to align sequencing data and authenticate true alignments, such as aMeta, have short comings due to their alignment method, MALT. However, the authors lack to discuss which of the steps that they implemented into bamdam are also present in aMeta, which ones are only present in bamdam, which ones are only present in aMeta. I think the readers of this manuscript would highly benefit from such an overview.

We appreciate the suggestion. We have now added a brief description of the authentication

metrics in aMeta vs bamdam (lines 294-296).

Here are some general comments regarding the code provided on GitHub:

- Code installation: currently, there are only very rudimentary installation instructions available on the GitHub page. The instructions are based on the Python package index PyPI and use "pip" to install these but the authors do not provide any information on which Python version is required to be installed. Furthermore, the repository has to be cloned manually and the rights of the major Python script have to be changed to make it an executable. This is not really novice user friendly. Instead, I would highly recommend to convert the script into a conda package to increase the usability and allow a single-line installation of the software.

We agree. We have now made the package available as a single-line install through pip, a widely

used Python package manager, and have added version requirements. We also attempted to make

it available on conda, but found that the hyperloglog package, a core bamdam dependency, was

also not available on conda. Fixing this is somewhat nontrivial, but we agree it is worth doing, and

commit to doing in the near future.

- Programming style: the whole suite of "bamdam" that consists of five main sub-commands is spread across 27 functions in a single file. The script comprises almost 1,500 lines of Python code and is very hard to review. I would suggest to modularise the code and split it into multiple files to increase the readability. Furthermore, adding some test cases that a user could run to test the software without requiring them to first run BWA/BowTie2 and then ngsLCA would be appreciated.

Thanks for this suggestion. Multiple test cases have now been made available for download in

a full tutorial at the bottom of the github page.

We appreciate the reviewer’s suggestion regarding modularization. While we understand the

value of splitting code into multiple files, in this particular case, the bamdam script was intention-

ally designed as a single cohesive file. The script is carefully organized, commented, and structured

with readability. Keeping it as a single file simplifies distribution, installation, and usage, particu-

larly given its set of closely related sub-commands, which are not easily split into separate sets of

functions. However, if the reviewer feels strongly, we would be willing to split it up.

Thank you for your time and consideration.

**Second round of view**

**Reviewer 1**

De Sanctis et al. propose a post-mapping toolkit bamdam, which serves complementary to “ngsLCA” and “metaDMG-cpp lca” for processing and authentication of aligned (with bowtie2 or BWA) reads in ancient metagenomic samples.

Both the toolkit and especially the manuscript have substantially improved since the initial version, and I believe the benefits of bamdam are much more clearly demonstrated now in the latest version. I especially appreciated the new Figure 1, and the Figure 2 which explains in details the process of filtering with respect to the DUST score and Duplicity. I also found the new bamdam tutorial in the github page very useful, and I was able to successfully run the bamdam commands on the test samples and other samples from my previous projects. Remarkably, bamdam was possible to run on a laptop even for large bam-alignments in agreement with what is reported in the Table 1 of the manuscript. The bamdam documentation at the github page was clearly written and easy to follow, the command lines ran without obvious problems in my tests. The inclusion of the damage-colored Krona plot is very interesting and useful, it should be appreciated by the ancient metagenomics community.

I thank the authors for addressing all my comments, and I appreciate their efforts to improve the toolkit and the clarity of the manuscript.

I have a few minor comments / suggestions:

1) I would suggest to replace “smiley” by “deamination” in the Figure 1. While widely used, I am not sure that “smiley” is a proper terminology and not slang.

2) As far as I understand, the Figure 2 (duplicity vs. dust score) is not currently produced by bamdam by default. Although it is quite straightforward to construct such a figure from the output of “bamdam compute”, I believe the community would greatly appreciate if this plot (in particular, with highlighted outlying taxa) would be output by bamdam for each sample by default (or at least by enabling a certain option-flag). I would really encourage the authors to add this option to the bamdam toolkit. Also, the damage-coloring (the gradient bar) is a bit confusing to me as I thought 5’ C-to-T frequency cannot be higher than 0.5 while the gradient bar shows this up to 1.0. I sthat the sum of Damage+1 and Damage-1? Could the colors be better explained?

3) I noticed that multi-threading is currently not supported in bamdam (at least in “bamdam shrink” and “bamdam compute”), which resulted in very long waiting times in my case (up to 24 hours for deep-sequenced samples). If possible, I would recommend to add this option.

4) I would appreciate to see more discussion why Danio, Cherax, Octopus etc. in the Figure 2 have high damage values while presumably being false-positive hits. I believe this has something to do with ancient low-complexity reads mapping but would like the authors to provide more details.

**Reviewer 3**

Summary of the work:

The authors present bamdam a post-mapping and post-LCA assignment toolkit designed for processing outputs specifically generated by ngsLCA or metaDMG. The toolkit includes a series of functions:

1. Bamdam shrink: significantly reduces BAM and LCA file sizes
2. Bamdam compute: generates authentication metrics and substitution tables
3. Bamdam plotdamage: Plot deamination patterns per taxon for multiple samples
4. Bamdam krona: Plots sample in a pie chart reflecting abundance and taxonomy in a deamination-based colour gradient
5. Bamdam plotbaminfo: Plots edit distance and mean read length distribution
6. Bamdam extract: Enables the extraction of reads assigned to a given taxon, including all lower-ranking taxa for downstream analyses such as phylogenetics.

This toolkit will probably meet the needs of users of ngsLCA and metaDMG incorporated in the Holi pipeline. It incorporates useful authentication metrics known from pipelines like HOPS and aMeta, and introduces a new metric (mean DUST score) into ancient metagenomics.

The code is available on GitHub and is well documented, with a helpful tutorial. However, the scope of the toolkit remains limited due to input constraints, lack of combined output, and implementation as a single Python script.

Strengths:

- bamdam provides an essential postprocessing toolkit for users of metagenomic BAM + LCA workflows using ngsLCA or metaDMG, where comparable tools were previously lacking.
- The shrink functionality is particularly useful in HPC settings where storage is a limiting factor.
- The tool replicates key authentication metrics in ancient metagenomics also implemented in pipelines like HOPS and aMeta (e.g. read count, deamination patterns, mean read length, GC content, unique k-mers, edit distance and PMD scores). Since the mapping step of those tools can take time and be computationally-demanding, labs have resorted to mapping with bowtie2 and LCA assessment and this tool adds the final postprocessing layer.
- It also provides similar plots like mismatch distribution (edit distance), deamination plot, read length distribution and abundance pie charts.
- It introduces metrics less commonly used in ancient metagenomics, such as mean DUST score and k-mer duplicity.
- The tutorial is pedagogical, intuitive, and well written, with clearly named parameters and example output.

Weaknesses:

- The toolkit only supports ngsLCA/metaDMG outputs, limiting accessibility for users of the other main LCA tool: sam2lca.
- Misuse of terminology when describing k-mer duplicity as a proxy for breadth or evenness of coverage. However, the k-mer duplicity is used in the result and discussion section rather as a flag for potential false positiveness. This description as proxy for breadth of coverage should be removed altogether. At best, from all the metrics provided, unique k-mers counts would be the most appropriate to be called as proxy for breadth of coverage since they indicate how many unique regions of a genome are covered at least once.
- Lack of integrated visual summary or authentication scoring which requires users to interpret several separate plots for many taxa.
- Lack of recommendation of specific metric threshold when using this toolkit for the isolation of likely true hits.
- Lacks a function to evaluate depth, breadth, or evenness of coverage from the output and to plot the coverage.
- PMD scores are computed but not plotted which is confusing. I recommend either plotting them or not computing them at all depending on the author’s interpretation of their usefulness.
- Main output BAM files from *bamdam extract* are multi-reference and not directly usable for typical downstream analyses. They also contain all the references headers, even the ones for which they are no read aligned for that specific taxa. I tried sorting and indexing the bam file generated by bamextract in the tutorial and encountered and error (Numerical result out of range).
- The implementation as a single, ~2000-line Python script can make the codebase more difficult to navigate and maintain. Consider modularising components or wrapping the tool in a workflow manager like Snakemake or Nextflow in the future.

Major comments:

- I strongly recommend extending support to LCA outputs from sam2lca, as this would make bamdam relevant to a broader range of users beyond the current ngsLCA/metaDMG ecosystem.
- Revise the misleading description of k-mer duplicity as a proxy for breadth or evenness of coverage; it would be more accurate to present it as an indicator of false positives, read stacking, or high clonality (the latter mostly relevant in capture enrichment contexts). Unique k-mers count has been used as a proxy for breadth of coverage and their ratio with the read count as a proxy for evenness of coverage (see minor comments directly on the text). It could be interesting to investigate further if k-mers are a useful value for the detection of true positives in bamdam.
- Introduce default threshold values or at least suggest threshold values for the newly introduced metrics in order to reduce false positive detection. Also mention any other threshold used in the toolkit functions (like read count in the shrink option if it is the case) and their default threshold values.
- On damage calculation and plotting:
  - Please clarify whether substitution profiles for a read are averaged across all alignments regardless of taxon, or only within alignments to a specific taxonomic node. Averaging across divergent taxa could artificially inflate damage estimates due to mismappings, particularly in metagenomic datasets.
  - The elevated baseline damage (~0.02) observed in modern, presumably undamaged samples in Figure 1 may be a result of this averaging strategy. If so, a stricter approach that avoids cross-taxon averaging could help reduce this artefact.
  - It would be helpful to include a schematic in the supplementary material showing how damage is computed in bamdam. A comparison with one established tool like mapDamage, PMDTools, or DamageProfiler on the same dataset would also help clarify whether the observed baseline is specific to bamdam.
  - It would be a nice addition to add a sample name legend to multi-sample damage plots. Currently there is no way of distinguishing which line is for which sample.
- The performance test on real data is a valuable addition, but it falls short of demonstrating two key points: (1) whether shrinking the BAM files still allows detection of the same species as in the original study, and (2) whether the added metrics help efficiently identify true ancient species compared to the original results.
- In the method section, the comparison with the full pipeline of aMeta is misleading, as aMeta does not produce BAM + LCA outputs and the pipeline failed at the MALT step, which doesn’t prevent the bowtie2 step to run successfully when using the parameter –keep-going on snakemake pipelines. A fair benchmark would use aMeta’s Bowtie2 mapping branch, apply the same database, and follow with LCA assignment and bamdam. However, this would be comparing mapping with Bowtie2 and mapping with Bowtie2 within aMeta which would not be very informative. I recommend removing or reframing this comparison to avoid conflating distinct pipeline goals and formats.

Recommendations:

- Please mention in the manuscript that bamdam extract includes a parameter to extract reads assigned only to the top reference of a node. This improves downstream compatibility by avoiding multi-reference BAM files, which are prone to follow-up errors.
- Although bamdam does not output depth, breadth, or evenness of coverage, these can be easily computed from the extracted top-reference BAM. However, since users may wish to include such plots as authentication evidence in their case studies, consider adding basic support or examples to the toolkit.
- Remove unused references from the header of multi-reference BAM files to simplify the files.
- PMD scores are currently computed but not plotted. Either integrate plotting support or omit the metric to avoid confusion.
- For Figure 2, consider adding zoomed-in views on regions with low duplicity and low DUST scores to highlight likely true positives. As it stands, the plot primarily illustrates how to exclude false positives, rather than how to detect genuine ancient taxa.
- Consider adding a summary report or overview plot combining most authentication metrics for a clearer overview of each organisms detected.

Terminology:

- The term least-common-ancestor is less common and less intuitive than its synonym: **lowest common ancestor (LCA)**. I recommend defining lowest common ancestor when first introduced, using the abbreviation LCA consistently thereafter, and removing hyphens for clarity.
- Define ***k*-mer duplicity** and **mean DUST score** when first mentioned with at least some words in brackets like: *k*-mer duplicity (average number of times each *k*-mer appears) and mean DUST score (read set complexity).
- In order not to loose the reader it would be ideal to speak of “***k*-mer duplicity”** and only use the word duplicity alone when it has been used not far away in the text.
- Make sure to use **only one term for a novel metric**. Since the DUST score already exists, I would stick to **mean DUST score** and only use “read set complexity” or “mean read complexity” or “read set *k*-mer based metric”to describe it, otherwise you have four synonyms for the same thing which makes it complicated.
- “Smiley” plot is too colloquial. They are most commonly called **deamination plots**, damage profiles, or one can speak of plotting deamination patterns or postmortem damage patterns or ancient dna damage patterns.
- Please verify how **tool names** are represented in their respective papers, and reuse that name format (capitalised, uncapitalised etc). Same applies to **file formats**.
- Note that the k of ***k*-mers** should be italicised.

Minor comments directly on the text:

Introduction:

The introduction is clear and well-written. I have some context-specific comments.

Line 47: There is no reason to cite wholeskim as an example of commonly used *k*-mer classifier methods since it is not even yet published and therefore not widely used. It would make more sense to cite Kraken2, on top of KrakenUniq, since it is widely used because much faster.

Line 64: “using KrakenUniq’s duplicity and evenness of coverage outputs”.
Please rephrase. KrakenUniq doesn’t output any column called duplicity or evenness of coverage. aMeta’s filters at that step are species level, read count and unique *k*-mers.

Line 68: The phrase “and an associated authentication method HOPS” could be misleading, as it suggests HOPS is part of aMeta. In fact, aMeta uses MALT and MaltExtract but not the full HOPS pipeline. I suggest rephrasing to: “MALT, or Megan Alignment Tool, is a fast and commonly used metagenomic aligner with a lowest common ancestor step. It is used in aMeta and also forms the core aligner in the HOPS pipeline [28].”

Line 72-73: I have doubts about the workaround mentioned for MALT. Please make sure that this is really possible if you want to keep that sentence. MALT doesn’t output common bam file that can simply be merged.

Lines 78–79: The statement “has lower accuracy in ancient metagenomic contexts [11]” should be nuanced. The version of MALT used for benchmarking in that study contained a bug that affected its accuracy, which was introduced in that version and later corrected in subsequent versions. <https://megan.cs.uni-tuebingen.de/t/lca-placement-failure-with-malt-v-0-5-2-and-0-5-3/1996>

Line 85: The phrase “is commonly referred to as the Holi pipeline” may overstate its recognition, given that the pipeline is unpublished. I recommend replacing it with “implemented in the Holi pipeline” for accuracy and neutrality.

Methods:

The method section is fairly short and I was a bit surprised that there was so little about the bamdam code, but in the end it is available on GitHub.

Line 332: The note that bamdam shrink and compute were run using a single thread is directly followed by aMeta’s high RAM usage, which could give the false impression that the overall bamdam workflow is not resource-intensive. If the aMeta RAM requirement is retained, please also provide an estimate of the RAM and clock time used during the Bowtie2 indexing and mapping steps and ngsLCA step to offer a fair comparison of resource demands.

Line 333: The authors mention difficulty running aMeta due to the MALT step’s high memory usage. However, MALT produces .rma6 files, not BAM, and is not relevant for benchmarking a tool like bamdam. For a fair comparison, they should have used aMeta’s Bowtie2 branch with the same reference database and then applied LCA and bamdam. Consider clarifying or removing completely this section.

Results

Line 98 (1^st^ result paragraph): can the authors please clarify or provide a range or percentage for what they call “order of magnitude”

Line 148 (bamshrink): The phrase “or which have too few reads assigned to them” is vague. Please specify the default read count threshold used for filtering when the user does not manually set this parameter.

Line 154 (bamshrink): The phrase “exclude user-defined taxonomic nodes” could be misleading, as it may suggest the user defines custom taxa. Please clarify whether this refers to removing specific nodes listed in the LCA file, and whether this functionality is directly implemented in bamshrink or simply recommended as a preprocessing step.

Line 158 (bamshrink): The term “similarity” is unclear in this context. Please specify what metric is used (e.g. alignment identity, edit distance, etc.) and what the default threshold is in the program when not explicitly set by the user.

Line 170: Please clarify whether the “total reads” value per node refers only to reads assigned directly to that node, or if it also includes reads assigned to all descendant (lower-ranking) taxa — for example, whether genus-level counts include species-level assignments.

Line 186 (bamdam compute): The sentence “the number of unique k-mers has also been shown to be useful as an authentication metric” could be expanded. Several studies have combined unique k-mer counts with other variables to derive more informative authentication scores. For example, Guellil et al. (2022, Genome Biology) used an e-value based on (K/R)×C, where K is the unique k-mer count, R is the read count, and C is the reference coverage (from KrakenUniq). Rodríguez-Varela et al. (2024, Science Advances) used a simpler K/R ratio as a proxy for evenness of coverage. I’d be interested to know if the authors have explored such combinations, and whether they might consider adding these values to the TSV output if found to be informative. These derived metrics could enhance

Line 236 (bamdam plotdamage): How can one distinguish which damage line is what? Is there a color scheme or a stroke style scheme? In figure one, it is not clear what taxonomic node or sample is represented by the multi-sample postmortem damage plot. By looking on the github one understands that the damage plots can be multi-sample but are *per taxon*. Please clarify in text.

Line 246 (bamdam krona): the plot on figure 1 seems according to the scale to be only based on the C-to-T misincorporation frequency. If the other metrics mentioned in this paragraph are also used to calculate that score, please specify how it is calculated per taxa. Also specify if the sample used for the figure was single or double-stranded.

Discussion:

Please compare how good bamdam plotting of the duplicity and mean DUST score is at providing valid authentication by comparing the results from Figure 2 for these three samples with the results from the original study for these three samples.

Also consider if there would be other plots on these real data that would best show the true hits and facilitate comparison with the original study’s results.

Could you also please briefly explain why a capture enrichment sample ERR10493315 (described as a capture enrichment sample in the legend of table 1), could contain so many different species hits in Figure 2. Please specify what was captured and consider using thresholds in order to show what was the likely captured sample and explain.

Figure 1:

- Figure 1 is a good graphical abstract of what bamdam actually does and can be used for.
- If possible, add the function bamdam combine to figure 1.
- Please specify in the legend if the library used for the damage plots was single-stranded or double-stranded.
- The damage-coloured Krona plots are a nice addition. However, I suggest inverting the colour scheme to make interpretation more intuitive. That means using red for highly damaged taxa and blue for low damage, following standard heatmap conventions.
- It is unclear from that figure how one can know which organism is which in the multi-sample deamination plots and that’s a disadvantage.

Figure 2

- Please clarify whether this figure is generated automatically by bamdam. If so, consider including it in the overview schematic in Figure 1 to improve clarity around the full workflow.
- Currently, the labelled species in Figure 2 tend to be those with high duplicity and/or high mean DUST scores, making them less relevant. To better highlight likely true positives, I suggest adding a zoomed-in view for each sample focusing on low-duplicity, low-DUST regions, and labelling taxa of interest there. Greyed-out or masked dots for undamaged hits could further help readers visually match the results to those reported in the original study.

Comments on GitHub:

- In the README, the suggested pip install method makes the ./ prefix unnecessary in commands. I recommend removing it to avoid confusing beginners. If alternative installation methods require a path, this could be clarified in a note.
- The TSV file headers are mostly clear, but more familiar naming for the two deamination sides would help. The RatioDupKmers column is less intuitive and should be briefly explained in the README or manuscript.
- It's unclear whether the TotalAlignments metric includes reads mapped to higher taxonomic nodes. Clarifying this would improve transparency.

Conclusion

Overall, bamdam is a promising and well-documented tool that fills a key gap for users of BAM + LCA workflows based on ngsLCA and metaDMG. With the suggested revisions, it could become an indispensable resource for ancient metagenomic authentication more broadly across similar workflows.

**Authors Response**

**Point-by-point responses to the reviewers’ comments:**

We greatly appreciate the second round of comments from an original reviewer, a new reviewer,

and the editor of Genome Biology, and are grateful for the chance to respond to these comments.

Our responses are in blue. By incorporating these comments, we think both the manuscript and

the program have improved greatly since the last version. We will first address the brief comments

from the editor.

As you will see, the reports are broadly favorable, so we are interested in publishing the

manuscript, but we feel that the issues raised must be addressed in full, in the form of a revised

manuscript, before we make a firm commitment to publication. When revising your manuscript,

please ensure that all the points raised by both referees are addressed. Please discuss the limitations

about supporting sam2lca outputs (Reviewer 2), as well as Reviewer 1’s suggestion to incorporate

multi-threading. However, we are not requiring an extension to support sam2lca outputs, though

we recognize it could be done in a future update.

Thank you very much. We agree that allowing sam2lca output as input to bamdam would be

beneficial. We do intend to implement this in a future update, as suggested. The difference between

sam2lca and ngsLCA is mainly in the extent of information in their outputs. Unlike ngsLCA, which

outputs full taxonomic paths per alignment, sam2lca outputs only a bam file with taxonomic node

IDs as bam tags for each alignment, which is not sufficient information to run the core functional-

ity of bamdam such as aggregation up the taxonomy. We will have to create an ngsLCA-style lca

file from the sam2lca bam output. But because the taxonomic paths are not in the sam2lca bam

file, the creation of this file would require taking all the taxonomy dump files as input (that is,

nodes.dmp, names.dmp and acc2tax) and parsing them to create an internal taxonomic structure.

This parsing is the main effort of both sam2lca and ngsLCA, both which warranted their own

software packages and publications, and is not so trivial.

We have now improved the speed substantially, so that bamdam shrink is about twice

as fast and bamdam compute is about 10x faster compared to the last round of revisions!

Because of this, we are now able to provide an additional benchmark: running bamdam shrink

and compute took less than a day and less than 20GB of RAM to process a 500GB input bam

file on a single thread. Certainly at this point, bamdam is neither a time nor RAM bottleneck in

its intended overall pipeline, as both the pairwise alignment step and the samtools merge/sort are

much more computationally demanding than running bamdam.

This is fortunate, because effectively multi-threading while retaining the current low RAM

overhead is nearly impossible, and in our view, the low RAM overhead is an important feature

of bamdam. In order to minimize RAM requirements, neither shrink nor compute will read the

entire bam file in RAM at any given time. Furthermore, the input bams are read-sorted, and so

cannot be indexed (and therefore threaded) easily as coordinate-sorted bams can be. In theory, we

can multithread bam decompression and compression, and indeed did try implementing this, but neither bamdam shrink nor compute are heavily I/O limited at this point, and so we found it did

not make a big difference in performance on real data.

Other than decompression and compression, we could consider threading the compute oper-

ations. Multithreading into n threads compute means a full initial read of the file for manual

indexing (time-consuming for large input), and then creation of 2n temporary bam and lca files: we

would have to read the entire input bam once to determine split points, then again to separately

write the input bam and lca into n unequal-sized chunks (unequal so as to not split alignments of

the same read across threads). Then each thread would take one of the n bam file chunks and its

associated lca file chunk, process it through bamdam compute, and send the node data back to

a central process which would combine all the separate instances of the node data object. Thus

each individual thread would consume nearly the amount of RAM as the entire process were it

running on one thread. This really does not seem worth it to us, plus the overhead will certainly

add nontrivial clock time. If the reviewer or editor sees a better way to do this which does not

dramatically compromise the low RAM usage, we are open to suggestions, but multi-threading

BAM operations is a known difficult task (e.g. samtools sort creates a large amount of temporary

sorted files and still consumes a huge amount of RAM).

We implemented a slightly different aggregation mode as default in bamdam compute. This ag-

gregation has implications for damage computation. This, and the multiple comments from review-

ers in both this and last round asking about the bamdam damage aggregation scheme, prompted

us to do a deep-dive into the different ways of computing damage in a metagenomic context. We

have added multiple figures on this now, and strongly suggest that this new, much faster mode is

also the most correct way to calculate postmortem damage in a metagenomic context. To this vein

and as per reviewer comments, we also extended the section looking at a real dataset substantially,

and a Supplement expaining the modes in detail.

**Reviewer 1**

De Sanctis et al. propose a post-mapping toolkit bamdam, which serves complementary to “ngsLCA” and “metaDMG-cpp lca” for processing and authentication of aligned (with bowtie2 or BWA) reads in ancient metagenomic samples.

Both the toolkit and especially the manuscript have substantially improved since the initial version, and I believe the benefits of bamdam are much more clearly demonstrated now in the latest version. I especially appreciated the new Figure 1, and the Figure 2 which explains in details the process of filtering with respect to the DUST score and Duplicity. I also found the new bamdam tutorial in the github page very useful, and I was able to successfully run the bamdam commands on the test samples and other samples from my previous projects. Remarkably, bamdam was possible to run on a laptop even for large bam-alignments in agreement with what is reported in the Table 1 of the manuscript. The bamdam documentation at the github page was clearly written and easy to follow, the command lines ran without obvious problems in my tests. The inclusion of the damage-colored Krona plot is very interesting and useful, it should be appreciated by the ancient metagenomics community.

I thank the authors for addressing all my comments, and I appreciate their efforts to improve the toolkit and the clarity of the manuscript.

We are very happy to hear that the reviewer has successfully ran bamdam on their own data,

and that they are satisfied with the edits. We agree that it is much improved with the incorporation

of Reviewer 1’s comments.

I have a few minor comments / suggestions:

1) I would suggest to replace “smiley” by “deamination” in the Figure 1. While widely used, I am not sure that “smiley” is a proper terminology and not slang.

We agree. This has been done.

2) As far as I understand, the Figure 2 (duplicity vs. dust score) is not currently produced by bamdam by default. Although it is quite straightforward to construct such a figure from the output of “bamdam compute”, I believe the community would greatly appreciate if this plot (in particular, with highlighted outlying taxa) would be output by bamdam for each sample by default (or at least by enabling a certain option-flag). I would really encourage the authors to add this option to the bamdam toolkit.

This is now an option included in bamdam compute by enabling an optional flag as suggested.

Also, the damage-coloring (the gradient bar) is a bit confusing to me as I thought 5’ C-to-T frequency cannot be higher than 0.5 while the gradient bar shows this up to 1.0. I sthat the sum of Damage+1 and Damage-1? Could the colors be better explained?

We find this is a common misconception in ancient DNA and are unsure where it originated

from. In a single-reference context (not metagenomic), as implemented in mapDamage, and to

our knowledge in every single-reference damage software since then, the 5’ C-to-T frequency is

measured as: the number of times we see a C-to-T transition over all reads, divided by the number

of times we see a C-to-anything (including C-to-C) over all reads. Thus, it only considers reads in

which the reference had a C there. This can theoretically go up to 1 in a single source context. As a

simple example, consider the scenario where we only have one read, and it has a C-to-T transition

on the 5’ end. This would be a 5’ C-to-T frequency of 1. In a metagenomic context things become

a bit more complicated, and we have added a discussion of this to the manuscript, but it can still

go up to 1 in theory. The Kap Kopenhagen paper (“A 2 million year old ecosystem revealed by

ancient DNA”) supplementary Fig. S6.4.2.2, for example, shows several damage values above 0.5.

3) I noticed that multi-threading is currently not supported in bamdam (at least in “bamdam shrink” and “bamdam compute”), which resulted in very long waiting times in my case (up to 24 hours for deep-sequenced samples). If possible, I would recommend to add this option.

Unfortunately multithreading bamdam compute is rather difficult, as discussed at the beginning

of this response. We aim to implement it in the future. In the meantime, we have made bamdam

much faster, and it should now take substantially less time on your samples.

4) I would appreciate to see more discussion why Danio, Cherax, Octopus etc. in the Figure 2 have high damage values while presumably being false-positive hits. I believe this has something to do with ancient low-complexity reads mapping but would like the authors to provide more details.

We agree and have added a more extensive discussion of this in the paragraph where DUST

scores are first introduced, and later in an updated section on the real data. We have realized that

the specific taxa depend substantially on the reference database used, and so this discussion no

longer specifically calls out Danio, Cherax and Octopus. Instead, we address the reviewer’s request

of providing details of ancient low-complexity read mappings more generally. In particular, we now

write :

‘No matter the origin, these low complexity reads are prone to map to similarly low-complexity

regions in reference genomes, and may even carry damage signals if they originated from a genuine

ancient source. If the true reference genome is not in the reference database, or the reference genome

is present but does not contain these regions – because low-complexity regions are especially difficult

to assemble – these reads may map elsewhere. Paradoxically, these reads may then be more likely

to spuriously map to high-quality reference genomes, as high-quality assemblies are more likely to

contain repetitive and low complexity regions. Taxonomic nodes for which read sets have a low

mean read complexity (i.e., high mean DUST score) are thus more likely to be artifacts, and this

issue may then become further exacerbated over time.’

and later, in reference to the dataset analyzed as a case study,

“The tendency of Metazoa families to have high DUST scores in this dataset reflects both the

composition of these samples and the composition of the reference database used for mapping.

We suspect that many of these Metazoa low-complexity identifications can be at least partially

attributed to genuinely ancient sequences but do not have true representative sequences in our

reference databases, and therefore spuriously map to a low-complexity region of a high-quality

Metazoa reference genome. RefSeq is rich in high-quality Metazoa reference genomes. Indeed,

many if not all of the families with high read count, high damage, and low complexity have at least

one chromosome-level assembly in RefSeq. ”

Danio, Cherax and Octopus all have chromosome-scale assemblies.

**Reviewer 3**

Summary of the work:

The authors present bamdam a post-mapping and post-LCA assignment toolkit designed for processing outputs specifically generated by ngsLCA or metaDMG. The toolkit includes a series of functions:

7. Bamdam shrink: significantly reduces BAM and LCA file sizes
8. Bamdam compute: generates authentication metrics and substitution tables
9. Bamdam plotdamage: Plot deamination patterns per taxon for multiple samples
10. Bamdam krona: Plots sample in a pie chart reflecting abundance and taxonomy in a deamination-based colour gradient
11. Bamdam plotbaminfo: Plots edit distance and mean read length distribution
12. Bamdam extract: Enables the extraction of reads assigned to a given taxon, including all lower-ranking taxa for downstream analyses such as phylogenetics.

This toolkit will probably meet the needs of users of ngsLCA and metaDMG incorporated in the Holi pipeline. It incorporates useful authentication metrics known from pipelines like HOPS and aMeta, and introduces a new metric (mean DUST score) into ancient metagenomics.

The code is available on GitHub and is well documented, with a helpful tutorial. However, the scope of the toolkit remains limited due to input constraints, lack of combined output, and implementation as a single Python script.

We appreciate the kind comments from reviewer 2, and have made many edits according to their

feedback below. There is now a bamdam combine function which addresses the ‘lack of combined

output’, and we have split the code into multiple organized scripts.

Strengths:

- bamdam provides an essential postprocessing toolkit for users of metagenomic BAM + LCA workflows using ngsLCA or metaDMG, where comparable tools were previously lacking.
- The shrink functionality is particularly useful in HPC settings where storage is a limiting factor.
- The tool replicates key authentication metrics in ancient metagenomics also implemented in pipelines like HOPS and aMeta (e.g. read count, deamination patterns, mean read length, GC content, unique k-mers, edit distance and PMD scores). Since the mapping step of those tools can take time and be computationally-demanding, labs have resorted to mapping with bowtie2 and LCA assessment and this tool adds the final postprocessing layer.
- It also provides similar plots like mismatch distribution (edit distance), deamination plot, read length distribution and abundance pie charts.
- It introduces metrics less commonly used in ancient metagenomics, such as mean DUST score and k-mer duplicity.
- The tutorial is pedagogical, intuitive, and well written, with clearly named parameters and example output.

Thank you!

Weaknesses:

- The toolkit only supports ngsLCA/metaDMG outputs, limiting accessibility for users of the other main LCA tool: sam2lca.

We agree. The editor has indicated that the journal is not requiring this extension at this

time, but we do intend to make bamdam compatible with sam2lca in a future update. Please

see the beginning of this response for details on why this is difficult.

- Misuse of terminology when describing k-mer duplicity as a proxy for breadth or evenness of coverage. However, the k-mer duplicity is used in the result and discussion section rather as a flag for potential false positiveness. This description as proxy for breadth of coverage should be removed altogether. At best, from all the metrics provided, unique k-mers counts would be the most appropriate to be called as proxy for breadth of coverage since they indicate how many unique regions of a genome are covered at least once.

As suggested, we removed the term ’breadth of coverage’ except to say that it is hard to

compute directly in a metagenomic context.

- Lack of integrated visual summary or authentication scoring which requires users to interpret several separate plots for many taxa.
- Lack of recommendation of specific metric threshold when using this toolkit for the isolation of likely true hits.

The reviewer brings up the issue of authentication scoring, thresholds, or summaries several

times in this review. This is an important point and we are grateful for this opportunity to

clarify our reasoning.

We have gone back and forth on whether or not to include thresholds or authentication

“scores”. In the end, after running bamdam on many of our own (unpublished) datasets, we

found that the threshold for all metrics is highly dataset-dependent – and differs tremendously

if the user used capture rather than shotgun sequencing.

Mindful of this variability, we decided to explicitly force users to specify their own thresholds

based on their familiarity with their own data sets. We believe this to be a feature that

encourages users to have a deeper familiarity with how bamdam functions and their own

ancient metagenomic datasets – bamdam was written with the hopes that it would encourage

a deeper exploration of ancient environmental DNA data. Other reviewers agree with us:

the requirement for users to interpret their own data was explicitly mentioned as a positive

by reviewer 2 in the first round of reviews. Furthermore, determining the threshold can be

accomplished quickly be examining the summary tsv tables, and does not typically require

inspecting several separate plots for many taxa as the reviewer suggests. All the relevant

information is included in the bamdam compute output tsv file(s), which can also be combined

into a single file using bamdam combine. This single, human-readable file, perhaps along with

the damage-coloured krona plots for easy visualization and data exploration, should therefore

be sufficient to make decisions (and has been in each of our own studies); each of the plotting

functions in bamdam is meant as an extra add-on visualization, and not as a necessary

component in deciding thresholds for the study.

We do now include the following line: “ “For example, the Prinseq documentation suggests

that scores over 7 can be considered low complexity”, and there are some example thresholds

set in the case study.

- Lacks a function to evaluate depth, breadth, or evenness of coverage from the output and to plot the coverage.

Coverage can only be meaningfully computed and plotted on a single reference genome, and

we feel that it does not belong in a metagenomics toolkit where most reads are assigned

to internal nodes and may map to thousands of reference genomes. However, in the github

tutorial, it is now shown how to use bamdam to easily plot coverage from bamdam output

on a reference genome of choice, such as the top-hit reference genome from the metagenomic

mapping. This top-hit reference genome ID itself is printed along with the bamdam extract

output when the –only-top-ref flag is used, and we now allow users to extract only the best

alignment, so that coverage plots can be generated immediately from the extracted file, eg

with samtools coverage -m.

- PMD scores are computed but not plotted which is confusing. I recommend either plotting them or not computing them at all depending on the author’s interpretation of their usefulness.

PMDTools are not computed by default – the user must manually add a flag for their in-

clusion. Computing them takes longer and we expect it to be usually be unnecessary. We

actually only added them because the original implementation in PMDTools has a major bug

in single-stranded mode (as mentioned in the github), so there is currently no other reliable

software that computes these scores. Although we do not consider them particularly impor-

tant in the context of bamdam, we feel that this is sufficient enough of a reason to leave

the implementation in as is, and hope the reviewer agrees. We are happy to remove them if

requested.

- Main output BAM files from *bamdam extract* are multi-reference and not directly usable for typical downstream analyses. They also contain all the references headers, even the ones for which they are no read aligned for that specific taxa. I tried sorting and indexing the bam file generated by bamextract in the tutorial and encountered and error (Numerical result out of range).

We fully agree with the reviewer this should be possible. Bamdam extract now does this by

default. Please see the updated tutorial.

- The implementation as a single, ~2000-line Python script can make the codebase more difficult to navigate and maintain. Consider modularising components or wrapping the tool in a workflow manager like Snakemake or Nextflow in the future.

We agree and have modularised the entire software into multiple separate files now.

Major comments:

- I strongly recommend extending support to LCA outputs from sam2lca, as this would make bamdam relevant to a broader range of users beyond the current ngsLCA/metaDMG ecosystem.

We agree and, as discussed directly with the editor, will add this to the software in the future.

- Revise the misleading description of k-mer duplicity as a proxy for breadth or evenness of coverage; it would be more accurate to present it as an indicator of false positives, read stacking, or high clonality (the latter mostly relevant in capture enrichment contexts). Unique k-mers count has been used as a proxy for breadth of coverage and their ratio with the read count as a proxy for evenness of coverage (see minor comments directly on the text). It could be interesting to investigate further if k-mers are a useful value for the detection of true positives in bamdam.

We note that a previous reviewer asked for us to refer to it as “breadth of coverage”, but have

now removed all references to breadth of coverage as requested, except to say it is difficult to

compute in a metagenomic context. As suggested, we also now do show the number of unique

k-mers is a useful value for the detection of true positives in an updated section looking at

real data.

- Introduce default threshold values or at least suggest threshold values for the newly introduced metrics in order to reduce false positive detection. Also mention any other threshold used in the toolkit functions (like read count in the shrink option if it is the case) and their default threshold values.

We have covered the first point above, and now mention flag thresholds in the manuscript.

- On damage calculation and plotting:
  - Please clarify whether substitution profiles for a read are averaged across all alignments regardless of taxon, or only within alignments to a specific taxonomic node. Averaging across divergent taxa could artificially inflate damage estimates due to mismappings, particularly in metagenomic datasets.

We understand the concern and have thought a lot about this point. Reads are assigned to

the least common ancestor of all their valid alignments, so substitution profiles are (necessar-

ily) computed using only the alignments to or below each taxonomic node (the latter of the

two options given by the reviewer). Substitution profiles are calculated per taxon, not per

read or per alignment.

In the updated bamdam compute function, we offer three computation modes: (1) using only

the best alignment per read, (2) averaging over reads only after they have been averaged

over their alignments, and (3) averaging over all alignments. In all cases, as stated above,

substitution profiles only consider reads assigned to that taxonomic node or below, which

will necessarily only have alignments assigned to that taxonomic node or below. In fact,

metaDMG uses the third mode, and we believe that it does indeed skew damage estimates

due to mismappings, which is largely mitigated by using mode (1) in bamdam (or even mode

2). We have added a discussion of the differences between different ways to compute damage

to the manuscript. We now recommend using only the best alignment per read.

- The elevated baseline damage (~0.02) observed in modern, presumably undamaged samples in Figure 1 may be a result of this averaging strategy. If so, a stricter approach that avoids cross-taxon averaging could help reduce this artefact.

We see the reviewer’s point. In this particular case, the elevated baseline damage is contextual:

the figure was generated from an environmental microbial metagenomics project where the

microbe in question did in fact have about 2% baseline divergence from its closest reference.

We now mention this in the figure caption to avoid confusion: ”the example in this figure

is for microbial samples prepared with single-stranded library prep, and which have a high

baseline divergence from their closest reference”. There are also now deamination plots on

the end for a few Viridiplantae. Thank you for pointing this out.

- It would be helpful to include a schematic in the supplementary material showing how damage is computed in bamdam. A comparison with one established tool like mapDamage, PMDTools, or DamageProfiler on the same dataset would also help clarify whether the observed baseline is specific to bamdam.

As we now offer multiple damage modes, we have also now added an extensive comparison

section and discussion of damage computation, as requested.

- It would be a nice addition to add a sample name legend to multi-sample damage plots. Currently there is no way of distinguishing which line is for which sample.

This is actually on purpose, as metagenomic experiments can have hundreds of samples and

labelling each line will render an entirely illegible figure. We embed an example here below in

Figure 1 on some real (unpublished) data we are working on. If there are few enough samples

that this is not an issue, the user can simply plot them as separate plots, as is typically done

in the supplement of many papers currently.

- The performance test on real data is a valuable addition, but it falls short of demonstrating two key points: (1) whether shrinking the BAM files still allows detection of the same species as in the original study, and (2) whether the added metrics help efficiently identify true ancient species compared to the original results.

The answers to your two points are a resounding yes and yes. The shrink function is described

in great detail in the manuscript. We have edited the language to clarify this further, and

added a short discussion and figure panel about it to the case study section. The shrinking

is designed to keep exactly and only those alignments and reads to the original species, genus

or family; ie. this is a yes to (1). And it has been documented extensively and repeatedly

in other studies that postmortem damage is an important metric for identifying true ancient

species; ie. this is a yes to (2). We have also now added a section showing the added metrics

help identify true ancient taxa.

- In the method section, the comparison with the full pipeline of aMeta is misleading, as aMeta does not produce BAM + LCA outputs and the pipeline failed at the MALT step, which doesn’t prevent the bowtie2 step to run successfully when using the parameter –keep-going on snakemake pipelines. A fair benchmark would use aMeta’s Bowtie2 mapping branch, apply the same database, and follow with LCA assignment and bamdam. However, this would be comparing mapping with Bowtie2 and mapping with Bowtie2 within aMeta which would not be very informative. I recommend removing or reframing this comparison to avoid conflating distinct pipeline goals and formats.

We agree and have accordingly removed these comments in the methods section.

Recommendations:

- Please mention in the manuscript that bamdam extract includes a parameter to extract reads assigned only to the top reference of a node. This improves downstream compatibility by avoiding multi-reference BAM files, which are prone to follow-up errors.

We agree and have now done this. The section reads: ’Bamdam extract extracts all

alignments from a bam file that belong to reads that have been assigned to a specific taxonomic

node(s) or below. This can be useful for downstream population genetic analyses such as

phylogenetic placement. The header of the output bam file is subsetted to only include

references which appear in the output bam file reads. Users may optionally also include only

the top reference and alignments to it.’

- Although bamdam does not output depth, breadth, or evenness of coverage, these can be easily computed from the extracted top-reference BAM. However, since users may wish to include such plots as authentication evidence in their case studies, consider adding basic support or examples to the toolkit.

We agree. An example demonstrating how to generate a coverage plot for the top ref is given

in the tutorial.

- Remove unused references from the header of multi-reference BAM files to simplify the files.

This is now default behavior in bamdam extract.

- PMD scores are currently computed but not plotted. Either integrate plotting support or omit the metric to avoid confusion.

This was addressed above.

- For Figure 2, consider adding zoomed-in views on regions with low duplicity and low DUST scores to highlight likely true positives. As it stands, the plot primarily illustrates how to exclude false positives, rather than how to detect genuine ancient taxa.

To this point, we now show damage-coloured Krona plots for the example published bams,

and added a discussion of detecting genuine ancient taxa in our example. The main purpose

of bamdam is to identify authentically ancient taxa and separate out false positives. We

expect that users will inspect output tables themselves to do this properly, and only intend

to illustrate the power of bamdam and some of our authentication metrics in the example

section.

- Consider adding a summary report or overview plot combining most authentication metrics for a clearer overview of each organisms detected.

We agree! This is exactly what bamdam combine does (in text form), and what bamdam

krona’s Summary output does (in plot form).

Terminology:

- The term least-common-ancestor is less common and less intuitive than its synonym: **lowest common ancestor (LCA)**. I recommend defining lowest common ancestor when first introduced, using the abbreviation LCA consistently thereafter, and removing hyphens for clarity.

This is true. We have fixed this.

- Define ***k*-mer duplicity** and **mean DUST score** when first mentioned with at least some words in brackets like: *k*-mer duplicity (average number of times each *k*-mer appears) and mean DUST score (read set complexity).

We have done this.

- In order not to loose the reader it would be ideal to speak of “***k*-mer duplicity”** and only use the word duplicity alone when it has been used not far away in the text.

Done.

- Make sure to use **only one term for a novel metric**. Since the DUST score already exists, I would stick to **mean DUST score** and only use “read set complexity” or “mean read complexity” or “read set *k*-mer based metric”to describe it, otherwise you have four synonyms for the same thing which makes it complicated.

Yes, we agree and have fixed this.

- “Smiley” plot is too colloquial. They are most commonly called **deamination plots**, damage profiles, or one can speak of plotting deamination patterns or postmortem damage patterns or ancient dna damage patterns.

Thanks, this has been changed to deamination plot throughout as suggested.

- Please verify how **tool names** are represented in their respective papers, and reuse that name format (capitalised, uncapitalised etc). Same applies to **file formats**.

As we understand the capitalization conventions, the software performs ngsLCA to perform

an LCA assignment and produce lca files. The software sam2lca does not produce output

files other than annotated bams. The typical way to refer to a bam file is in lowercase. We

have now made every mention match this.

- Note that the k of ***k*-mers** should be italicised.

Done.

Minor comments directly on the text:

Introduction:

The introduction is clear and well-written. I have some context-specific comments.

Line 47: There is no reason to cite wholeskim as an example of commonly used *k*-mer classifier methods since it is not even yet published and therefore not widely used. It would make more sense to cite Kraken2, on top of KrakenUniq, since it is widely used because much faster.

This is fair. We have changed this to cite Kraken2 instead.

Line 64: “using KrakenUniq’s duplicity and evenness of coverage outputs”.
Please rephrase. KrakenUniq doesn’t output any column called duplicity or evenness of coverage. aMeta’s filters at that step are species level, read count and unique *k*-mers.

The KrakenUniq paper, in the caption of Figure 1, states ’The output includes the number

of reads, unique k-mers, duplicity (average time each k-mer has been seen), and coverage for

each taxon observed in the input data’. Still, we have changed our phrasing.

Line 68: The phrase “and an associated authentication method HOPS” could be misleading, as it suggests HOPS is part of aMeta. In fact, aMeta uses MALT and MaltExtract but not the full HOPS pipeline. I suggest rephrasing to: “MALT, or Megan Alignment Tool, is a fast and commonly used metagenomic aligner with a lowest common ancestor step. It is used in aMeta and also forms the core aligner in the HOPS pipeline [28].”

Thanks, we have changed it as suggested.

Line 72-73: I have doubts about the workaround mentioned for MALT. Please make sure that this is really possible if you want to keep that sentence. MALT doesn’t output common bam file that can simply be merged.

We have removed that sentence.

Lines 78–79: The statement “has lower accuracy in ancient metagenomic contexts [11]” should be nuanced. The version of MALT used for benchmarking in that study contained a bug that affected its accuracy, which was introduced in that version and later corrected in subsequent versions. <https://megan.cs.uni-tuebingen.de/t/lca-placement-failure-with-malt-v-0-5-2-and-0-5-3/1996>

We have now changed it to ”may have”, as we could not find any further studies comparing

the two.

Line 85: The phrase “is commonly referred to as the Holi pipeline” may overstate its recognition, given that the pipeline is unpublished. I recommend replacing it with “implemented in the Holi pipeline” for accuracy and neutrality.

This has been changed as suggested.

Methods:

The method section is fairly short and I was a bit surprised that there was so little about the bamdam code, but in the end it is available on GitHub.

Line 332: The note that bamdam shrink and compute were run using a single thread is directly followed by aMeta’s high RAM usage, which could give the false impression that the overall bamdam workflow is not resource-intensive. If the aMeta RAM requirement is retained, please also provide an estimate of the RAM and clock time used during the Bowtie2 indexing and mapping steps and ngsLCA step to offer a fair comparison of resource demands.

We agree this is not a fair comparison and we have now removed the line.

Line 333: The authors mention difficulty running aMeta due to the MALT step’s high memory usage. However, MALT produces .rma6 files, not BAM, and is not relevant for benchmarking a tool like bamdam. For a fair comparison, they should have used aMeta’s Bowtie2 branch with the same reference database and then applied LCA and bamdam. Consider clarifying or removing completely this section.

We understand and have removed the line.

Results

Line 98 (1^st^ result paragraph): can the authors please clarify or provide a range or percentage for what they call “order of magnitude”

We have changed this to ”often by a factor of 10x or more”, also in the abstract.

Line 148 (bamshrink): The phrase “or which have too few reads assigned to them” is vague. Please specify the default read count threshold used for filtering when the user does not manually set this parameter.

Thanks, we have added the default parameter which is 5 reads.

Line 154 (bamshrink): The phrase “exclude user-defined taxonomic nodes” could be misleading, as it may suggest the user defines custom taxa. Please clarify whether this refers to removing specific nodes listed in the LCA file, and whether this functionality is directly implemented in bamshrink or simply recommended as a preprocessing step.

The user may in fact define custom taxa, as you correctly infer. The taxa have numeric node

IDs. We have rewritten this paragraph to clarify, and it now says:

”To perform the shrink function, bamdam runs through the lca file twice: once to determine

which taxonomic nodes pass filtering criteria, and again to extract reads assigned to those

nodes. This initial filtering removes reads in the lca file assigned to taxonomic nodes which

are above the taxonomic rank threshold (“family” by default) or which have too few reads

assigned to them (5 reads by default). In addition to these flags, the shrink step is configurable

in several ways. First, the user may manually pre-filter their lca file beforehand, as long as

reads are kept in the same order – for example, one might wish to remove all bacterial reads

before running bamdam. Second, the shrink step includes a (non-default) option to exclude a

custom, user-defined list of taxonomic nodes from the output shrunken bam and lca files, in

which case the reads are still aggregated up the taxonomy after these reads are excluded. For

example, it might sometimes be desirable to remove all taxonomic nodes from the samples

which were found in the negative controls as a stringent decontamination step. In this case

the user would run bamdam shrink first on the controls, extract the taxonomic node IDs, and

pass these into bamdam shrink to remove those nodes in the samples. Regardless of whether

the user excludes nodes or not, once the filtered lca file has been written, the bam will then

be filtered to only retain alignments associated to reads in the filtered lca file, and with more

than a user-defined similarity (the number of matches to the reference divided by the read

length; .9 by default). This yields a shrunken output bam file. The output bam can also

optionally be annotated with PMD scores as from PMDTools (off by default), although this

is more time-consuming on large bams. Running shrink is required before running compute.

Line 158 (bamshrink): The term “similarity” is unclear in this context. Please specify what metric is used (e.g. alignment identity, edit distance, etc.) and what the default threshold is in the program when not explicitly set by the user.

We agree this was unclear. It refers to the number of matches to the reference divided by the

read length. This is now in the manuscript (see our last response above, where we added this

to the paragraph).

Line 170: Please clarify whether the “total reads” value per node refers only to reads assigned directly to that node, or if it also includes reads assigned to all descendant (lower-ranking) taxa — for example, whether genus-level counts include species-level assignments.

We agree and have changed ”total reads” to ”total reads assigned to that node or below”.

Line 186 (bamdam compute): The sentence “the number of unique k-mers has also been shown to be useful as an authentication metric” could be expanded. Several studies have combined unique k-mer counts with other variables to derive more informative authentication scores. For example, Guellil et al. (2022, Genome Biology) used an e-value based on (K/R)×C, where K is the unique k-mer count, R is the read count, and C is the reference coverage (from KrakenUniq). Rodríguez-Varela et al. (2024, Science Advances) used a simpler K/R ratio as a proxy for evenness of coverage. I’d be interested to know if the authors have explored such combinations, and whether they might consider adding these values to the TSV output if found to be informative. These derived metrics could enhance

This is a good point. We now show later in the manuscript that the number of unique k -mers

does in fact help with filtering.

Let us discuss this using the reviewer’s own parameter names: K for the unique k-mer count,

R for the read count, and let us introduce J for the number of total k-mers, all for a given

taxonomic node in this context. Let us also introduce l as the mean read length for a

taxonomic node. Let’s use k=29 here, but note that this could easily be replaced with any

other value for k.

If the mean read length is l, the average read has (l-29) kmers. So the R total reads would

have approximately

J = R ∗ (l − 29)

total kmers. This is approximate because in reality we would have to integrate over the full

read length distribution, but it is a sufficient approximation for our purposes here. This

means that duplicity is calculating

J/K ≈ K

R(l − 29)

Since 0 < K ≤ J, the duplicity J/K is always at least 1, and is exactly 1 in the perfect

scenario where every k-mer is unique. We find this easy to understand: it is simply asking,

how much was the average k-mer duplicated?

On the other hand, the Rodr´ıguez-Varela et al. ratio K/R is essentially a scalar multiple of

duplicity (by a factor of approximately 1/(l − 29) in this case), but does not benefit from

this simple interpretation. However, if a user was interested in this specifically, it is easy to

calculate from the existing bamdam output, as both the total number and unique number of

k-mers is included in the tsv files generated. The e-value (K/R)xC where C is the reference

coverage is another option brought up by the reviewer, but in our case we cannot calculate

reference coverage, because there is not a single reference to use in this metagenomic context.

Line 236 (bamdam plotdamage): How can one distinguish which damage line is what? Is there a color scheme or a stroke style scheme? In figure one, it is not clear what taxonomic node or sample is represented by the multi-sample postmortem damage plot. By looking on the github one understands that the damage plots can be multi-sample but are *per taxon*. Please clarify in text.

This was addressed above!

Line 246 (bamdam krona): the plot on figure 1 seems according to the scale to be only based on the C-to-T misincorporation frequency. If the other metrics mentioned in this paragraph are also used to calculate that score, please specify how it is calculated per taxa. Also specify if the sample used for the figure was single or double-stranded.

Other metrics are not used, which is why the legend title is Damage. We have now specified

that the sample is single-stranded.

Discussion:

Please compare how good bamdam plotting of the duplicity and mean DUST score is at providing valid authentication by comparing the results from Figure 2 for these three samples with the results from the original study for these three samples.

Also consider if there would be other plots on these real data that would best show the true hits and facilitate comparison with the original study’s results.

Could you also please briefly explain why a capture enrichment sample ERR10493315 (described as a capture enrichment sample in the legend of table 1), could contain so many different species hits in Figure 2. Please specify what was captured and consider using thresholds in order to show what was the likely captured sample and explain.

We have now greatly expanded this section, as suggested, and hope that we succeeded in the

reviewer’s request of ”other plots on these real data that would best show the true hits and

comparison with the original study’s results”.

Figure 1:

- Figure 1 is a good graphical abstract of what bamdam actually does and can be used for.

Thank you.

- If possible, add the function bamdam combine to figure 1.

Thanks, this has been done.

- Please specify in the legend if the library used for the damage plots was single-stranded or double-stranded.

This has been done (single-stranded).

- The damage-coloured Krona plots are a nice addition. However, I suggest inverting the colour scheme to make interpretation more intuitive. That means using red for highly damaged taxa and blue for low damage, following standard heatmap conventions.

We see the reviewer’s point. Unfortunately, as bamdam has existed for over a year, these

damage-coloured krona plots have already been made public and submitted as part of unpub-

lished studies in more than one instance. We know of multiple studies in which bamdam is

being used, and indeed one in which it has been submitted and in which the current authors

are not involved. Therefore, we feel that inverting the colour scheme at this point will only

lead to more confusion downstream.

- It is unclear from that figure how one can know which organism is which in the multi-sample deamination plots and that’s a disadvantage.

Each line is a sample, not an organism. This comment was already made and has been

addressed above. We have also clarified this on both the github, main text and figure captions.

We also hope it is clear because the plots auto-generate a title with the (one) taxa name.

Figure 2

- Please clarify whether this figure is generated automatically by bamdam. If so, consider including it in the overview schematic in Figure 1 to improve clarity around the full workflow.

This figure was not automatically generated by bamdam during the previous submission, but

following both this comment and a comment from Reviewer 1 requesting that we add it as

an option to bamdam compute, we have done exactly this.

- Currently, the labelled species in Figure 2 tend to be those with high duplicity and/or high mean DUST scores, making them less relevant. To better highlight likely true positives, I suggest adding a zoomed-in view for each sample focusing on low-duplicity, low-DUST regions, and labelling taxa of interest there. Greyed-out or masked dots for undamaged hits could further help readers visually match the results to those reported in the original study.

We have instead overhauled the plot, in part due to Reviewer 2’s comments such as this one,

and it now better highlights likely true positives.

Comments on GitHub:

- In the README, the suggested pip install method makes the ./ prefix unnecessary in commands. I recommend removing it to avoid confusing beginners. If alternative installation methods require a path, this could be clarified in a note.

You’re right, thanks, this has been changed now.

- The TSV file headers are mostly clear, but more familiar naming for the two deamination sides would help. The RatioDupKmers column is less intuitive and should be briefly explained in the README or manuscript.

Thanks, there is a brief description of RatioDupKmers in the README: ”Another way of

thinking about duplicity: 1 minus the ratio of unique k-mers divided by the number of total

k-mers. Should be close to 0 (equivalent to no duplicated k-mers) unless coverage is high or

breadth of coverage is uneven.”

- It's unclear whether the TotalAlignments metric includes reads mapped to higher taxonomic nodes. Clarifying this would improve transparency.

We agree. The TotalAlignments in the README is now described as ”TotalAlignments:

Sum of the number of alignments for all the reads assigned to that node or underneath.”

Conclusion

Overall, bamdam is a promising and well-documented tool that fills a key gap for users of BAM + LCA workflows based on ngsLCA and metaDMG. With the suggested revisions, it could become an indispensable resource for ancient metagenomic authentication more broadly across similar workflows.

Thank you for your time and consideration.

**Third round of review**

**Reviewer 1**

I thank the authors for addressing my questions and comments. Well done! I believe, the manuscript and the tool improved a lot during the review process.

My final comment: if bamdam could compute damage pattern in UDG treated samples via calculating deamination signal at the CpG sites similar to the functionality available in PMDtools, this would be much appreciated by the ancient metagenomics community, in my opinion, and I really encourage the authors to implement this functionality within the bamdam tool.

**Reviewer 2**

This is an excellent and much-improved manuscript. My remaining points are minor corrections and clarifications. I therefore recommend acceptance pending minor revision. The authors have responded thoroughly to my questions and have substantially modified the manuscript, making the presentation of the work, the tool, and the real data application much clearer, which was the main focus of my previous review. I hope they also feel that my comments contributed to these improvements.

The explanations of duplicity, unique k-mers, and breadth of coverage are now clear, with a stronger emphasis placed on the role of unique k-mers. They have corrected all terminology inconsistencies I had noted and added clarifications in several places where the text was previously confusing or ambiguous. I also appreciate that they explained when some of my earlier expectations were not directly applicable here.

They have reflected extensively on my concerns regarding the computation of post-mortem damage across taxa, and I believe the resulting benchmarking will be valuable to the broader metagenomics field and likely referred to in future work.

They have removed the unfair comparison with full pipelines, which is appropriate since bamdam operates at the end of a mapping + ngsLCA workflow. They now even suggest in the manuscript that bamdam could be implemented after the aMeta Bowtie2 branch, which would make it even more useful.

Although they refrain from proposing universal thresholds across study types, they now show more clearly in the real data example how one could approach thresholding and provide some indicative values. The corresponding plots are also easier to interpret at first glance.

On the coding side, bamdam has been modularised, making the repository easier to navigate on GitHub.

Shrink function: The authors have improved the description of how to use bamshrink in the manuscript.

Extract function: They have enabled users to extract only the best alignments for downstream analyses and clarified the function’s logic. They also corrected the BAM headers, which previously prevented beginners from using the extracted files directly.

Krona function: The authors have convincingly explained why changing the colour scheme would be problematic, as the existing one is already used in other studies. I agree that consistency is preferable and that the community will adapt.

Compute function: It is a nice improvement that Figure 1 has now been integrated into this function, allowing beginners to easily visualise and compare their results with those shown in the bamdam paper.

Remaining points

1. k-mer/reads ratio and equation correction

I was not entirely convinced by the explanation regarding the k-mer/reads ratio in the response to reviewers. From equation 1 to 2, if we divide by K on the left, we should also divide by K on the right, rather than have K divided by what was there.

(correction of equation 2)

This means that K/R is inversely proportional (up to a constant scaling factor of roughly l – 29) to the duplicity. If we replace the k-mer length of 29 by k, we obtain this equation:

That relationship is precisely what makes the k-mers/reads ratio a good proxy for evenness of coverage, because it increases with evenness of coverage and decreases with read stacking.

I therefore strongly recommend that the authors implement this ratio as a computing option in bamdam and mention it in the manuscript as the ratio has already been used in previous studies (e.g. Guellil et al. 2022; Rodríguez-Varela et al. 2024). I agree that duplicity is always at least 1 and reflects how much k-mers are duplicated. Duplicity is thus a good proxy for read stacking, and low duplicity indicates low stacking, which corresponds to evenness of coverage, but it is inversely proportional. These are simply different ways of expressing the same relationship, using variables that are intrinsically linked. It is reassuring that several scholars have reached similar conclusions that the HyperLogLog algorithm and unique k-mers are very valuable for distinguishing true from false positives.

2. Definition of RatioDupKmers in the README

The current README states:

RatioDupKmers: Another way of thinking about duplicity: 1 minus the ratio of unique k-mers divided by the number of total k-mers. Should be close to 0 (equivalent to no duplicated k-mers) unless coverage is high or breadth of coverage is uneven.

However, based on the equation provided above, duplicity is J/K not K/J, so the description should be corrected accordingly. I suggest rephrasing it as:

RatioDupKmers: Same as duplicity but offset by 1, so it starts from 0 instead of 1.

3. Clarification of the aMeta filtering step

The revised phrasing at line 60 still implies that aMeta filters results using “duplicity” and “evenness of coverage.” In fact, the filters applied at that stage are to use only the species taxonomic level, the number of reads, and the number of unique k-mers.

A minimal correction would therefore be:

“This two-step process allows the early exclusion of some potential database contaminants beforehand using KrakenUniq’s unique k-mers.”

4. “Order of magnitude” phrasing

The correction from “order of magnitude” to “often by a factor of 10× or more” was only applied in the abstract. The same wording should also be used consistently under Figure 1 and in the discussion for clarity.

5. Clarifying the MALT version used in benchmarking

Although this is not something the authors can fully control, it would be more informative if the manuscript specified that the benchmarking study compared MALT **v0.5.3** (as listed under “Software versions” in the cited reference). If the editor allows, I recommend referring to the release_notes.txt of the software to note that a bug preventing the LCA algorithm from being applied to taxonomic classifications was fixed from v0.6.0 onwards (https://software-ab.cs.uni-tuebingen.de/download/malt/release_notes.txt).

MALT is a widely used tool, and while a benchmarking study is valuable, it does not reflect the normal behaviour of more recent, corrected versions.

6. DUST score filtering option

Please consider implementing a parameter that allows DUST score filtering within bamdam. The mean DUST score is very useful, but in cases where the reference genome contains many low-complexity regions, the overall mean dust score can become excessively high. This does not necessarily mean that no genuine signal is present. Sometimes a true hit may lie beneath the noise.

Allowing users to set a custom DUST score threshold would make it possible to remove the lowest-complexity reads and then assess whether enough reads remain for a given taxon to warrant further investigation. This suggestion stems from discussions with colleagues who are already using bamdam.

7. Quick fixes in the supplementary information

Please address the following minor corrections in the supplementary material:

A missing reference is indicated in the text:

“This behaviour is also similar to that of some pairwise aligners, e.g. if there are multiple best alignments, Bowtie2 will report a random one by default [? ].”

Also, before Equation 3, it could be helpful to briefly remind the reader that T-to-T transitions are irrelevant to the computation of C-to-T frequencies. For example, a short clarifying sentence such as “Note that T-to-T mismatches are not considered in the C-to-T frequency calculation as described above” would make the reasoning easier to follow in the given example.

Overall, I strongly recommend that the authors address these final points. Otherwise, this is an excellent tool, already being adopted by the community, and it certainly deserves publication soon. Great job!

**Authors Response**

**Point-by-point responses to the reviewers’ comments:**

**Reviewer 1**

I thank the authors for addressing my questions and comments. Well done! I believe, the manuscript and the tool improved a lot during the review process.

My final comment: if bamdam could compute damage pattern in UDG treated samples via calculating deamination signal at the CpG sites similar to the functionality available in PMDtools, this would be much appreciated by the ancient metagenomics community, in my opinion, and I really encourage the authors to implement this functionality within the bamdam tool.

Thank you very much. Your comments helped the manuscript and the software a lot. The UDG treatment mode is a great idea and we have now implemented it as a flag in bamdam compute.

**Reviewer 2**

This is an excellent and much-improved manuscript. My remaining points are minor corrections and clarifications. I therefore recommend acceptance pending minor revision. The authors have responded thoroughly to my questions and have substantially modified the manuscript, making the presentation of the work, the tool, and the real data application much clearer, which was the main focus of my previous review. I hope they also feel that my comments contributed to these improvements.

The explanations of duplicity, unique k-mers, and breadth of coverage are now clear, with a stronger emphasis placed on the role of unique k-mers. They have corrected all terminology inconsistencies I had noted and added clarifications in several places where the text was previously confusing or ambiguous. I also appreciate that they explained when some of my earlier expectations were not directly applicable here.

They have reflected extensively on my concerns regarding the computation of post-mortem damage across taxa, and I believe the resulting benchmarking will be valuable to the broader metagenomics field and likely referred to in future work.

They have removed the unfair comparison with full pipelines, which is appropriate since bamdam operates at the end of a mapping + ngsLCA workflow. They now even suggest in the manuscript that bamdam could be implemented after the aMeta Bowtie2 branch, which would make it even more useful.

Although they refrain from proposing universal thresholds across study types, they now show more clearly in the real data example how one could approach thresholding and provide some indicative values. The corresponding plots are also easier to interpret at first glance.

On the coding side, bamdam has been modularised, making the repository easier to navigate on GitHub.

Shrink function: The authors have improved the description of how to use bamshrink in the manuscript.

Extract function: They have enabled users to extract only the best alignments for downstream analyses and clarified the function’s logic. They also corrected the BAM headers, which previously prevented beginners from using the extracted files directly.

Krona function: The authors have convincingly explained why changing the colour scheme would be problematic, as the existing one is already used in other studies. I agree that consistency is preferable and that the community will adapt.

Compute function: It is a nice improvement that Figure 1 has now been integrated into this function, allowing beginners to easily visualise and compare their results with those shown in the bamdam paper.

Thank you! Your comments certainly did help a lot to improve both the manuscript and the software.

Remaining points

1. k-mer/reads ratio and equation correction

I was not entirely convinced by the explanation regarding the k-mer/reads ratio in the response to reviewers. From equation 1 to 2, if we divide by K on the left, we should also divide by K on the right, rather than have K divided by what was there.

(correction of equation 2)

This means that K/R is inversely proportional (up to a constant scaling factor of roughly l – 29) to the duplicity. If we replace the k-mer length of 29 by k, we obtain this equation:

That relationship is precisely what makes the k-mers/reads ratio a good proxy for evenness of coverage, because it increases with evenness of coverage and decreases with read stacking.

I therefore strongly recommend that the authors implement this ratio as a computing option in bamdam and mention it in the manuscript as the ratio has already been used in previous studies (e.g. Guellil et al. 2022; Rodríguez-Varela et al. 2024). I agree that duplicity is always at least 1 and reflects how much k-mers are duplicated. Duplicity is thus a good proxy for read stacking, and low duplicity indicates low stacking, which corresponds to evenness of coverage, but it is inversely proportional. These are simply different ways of expressing the same relationship, using variables that are intrinsically linked. It is reassuring that several scholars have reached similar conclusions that the HyperLogLog algorithm and unique k-mers are very valuable for distinguishing true from false positives.

Oops – yes, we certainly meant to have written

K/J = K/ (R(l-k))

Which is what you wrote above. Thanks for noticing that and for going into this in depth.

In the bamdam code, we don’t do any of this math – we simply sum up the number of unique k-mers and the number of total k-mers per node, and then do the latter divided by the former (J/K = duplicity). So luckily this has been implemented correctly despite our math typo in the previous revision round.

In any case, we now implement K/R (unique k-mers / total reads) ratio in the output, as requested, and cite the studies mentioned.

Line 172 now reads:

“The number of unique \emph{k}-mers has been shown to be useful as an authentication metric, as has the number of unique \emph{k}-mers divided by the read count \citep{Oskolkov20252,ameta,RodrguezVarela2024}”

2. Definition of RatioDupKmers in the README

The current README states:

RatioDupKmers: Another way of thinking about duplicity: 1 minus the ratio of unique k-mers divided by the number of total k-mers. Should be close to 0 (equivalent to no duplicated k-mers) unless coverage is high or breadth of coverage is uneven.

However, based on the equation provided above, duplicity is J/K not K/J, so the description should be corrected accordingly. I suggest rephrasing it as:

RatioDupKmers: Same as duplicity but offset by 1, so it starts from 0 instead of 1.

RatioDupKmers was here because of someone else’s request. It seems people have different intuitions that are more suited to different ratios, as pointed out above and evident from our correspondence. We do feel it is rather overkill to print three ratios measuring more or less the same thing (duplicity, K/R as requested above, and RatioDupKmers), especially when they can be easily determined from each other and the other existing outputs. For example, as the reviewer points out, RatioDupKmers is simply offset from duplicity. The requested ratio K/R is also a simple ratio of existing output (both K and R are output). In any case, we have removed RatioDupKmers and replaced it with K/R instead, as suggested, which we term UniqKmersPerRead in the output.

3. Clarification of the aMeta filtering step

The revised phrasing at line 60 still implies that aMeta filters results using “duplicity” and “evenness of coverage.” In fact, the filters applied at that stage are to use only the species taxonomic level, the number of reads, and the number of unique k-mers.

A minimal correction would therefore be:

“This two-step process allows the early exclusion of some potential database contaminants beforehand using KrakenUniq’s unique k-mers.”

Thanks, we have corrected this as suggested.

4. “Order of magnitude” phrasing

The correction from “order of magnitude” to “often by a factor of 10× or more” was only applied in the abstract. The same wording should also be used consistently under Figure 1 and in the discussion for clarity.

Good point, thanks, this has been corrected.

5. Clarifying the MALT version used in benchmarking

Although this is not something the authors can fully control, it would be more informative if the manuscript specified that the benchmarking study compared MALT **v0.5.3** (as listed under “Software versions” in the cited reference). If the editor allows, I recommend referring to the release_notes.txt of the software to note that a bug preventing the LCA algorithm from being applied to taxonomic classifications was fixed from v0.6.0 onwards (https://software-ab.cs.uni-tuebingen.de/download/malt/release_notes.txt).

MALT is a widely used tool, and while a benchmarking study is valuable, it does not reflect the normal behaviour of more recent, corrected versions.

This makes sense. We were not fully aware of this and do not mean to misrepresent MALT. To avoid all of this confusion, we have simply removed the part of the sentence “[MALT…] may have lower accuracy in metagenomic contexts”.

6. DUST score filtering option

Please consider implementing a parameter that allows DUST score filtering within bamdam. The mean DUST score is very useful, but in cases where the reference genome contains many low-complexity regions, the overall mean dust score can become excessively high. This does not necessarily mean that no genuine signal is present. Sometimes a true hit may lie beneath the noise.

Allowing users to set a custom DUST score threshold would make it possible to remove the lowest-complexity reads and then assess whether enough reads remain for a given taxon to warrant further investigation. This suggestion stems from discussions with colleagues who are already using bamdam.

It’s great to hear your colleagues are already using the software! We fully agree with this logic. We have now implemented a DUST score threshold in the shrink step, and mention it briefly at the end of the DUST paragraph in the manuscript. It was not immediately obvious whether this filter belongs in the shrink or the compute step, but choosing the latter would mean rewriting the extract step to use the same DUST filter (as it operates on the shrink-output bam and lca), which feels unintuitive, so we chose the former.

7. Quick fixes in the supplementary information

Please address the following minor corrections in the supplementary material:

A missing reference is indicated in the text:

“This behaviour is also similar to that of some pairwise aligners, e.g. if there are multiple best alignments, Bowtie2 will report a random one by default [? ].”

Also, before Equation 3, it could be helpful to briefly remind the reader that T-to-T transitions are irrelevant to the computation of C-to-T frequencies. For example, a short clarifying sentence such as “Note that T-to-T mismatches are not considered in the C-to-T frequency calculation as described above” would make the reasoning easier to follow in the given example.

Good catch. Thanks, we have fixed both of these things.

Overall, I strongly recommend that the authors address these final points. Otherwise, this is an excellent tool, already being adopted by the community, and it certainly deserves publication soon. Great job!

Thank you very much for your useful feedback!
